# Does global health governance walk the talk? Gender representation in World Health Assemblies, 1948–2021

**DOI:** 10.1136/bmjgh-2022-009312

**Published:** 2022-08-23

**Authors:** Kim Robin van Daalen, Maisoon Chowdhury, Sara Dada, Parnian Khorsand, Salma El-Gamal, Galiya Kaidarova, Laura Jung, Razan Othman, Charlotte Anne O'Leary, Henry Charles Ashworth, Anna Socha, Dolapo Olaniyan, Fajembola Temilade Azeezat, Siwaar Abouhala, Toyyib Abdulkareem, Roopa Dhatt, Dheepa Rajan

**Affiliations:** 1 Cardiovascular Epidemiology Unit, Department of Public Health and Primary Care, Cambridge University, Cambridge, UK; 2 Women in Global Health, Washington, District of Columbia, USA; 3 UCD Centre for Interdisciplinary Research, Education and Innovation in Health Systems, School of Nursing, Midwifery and Health Systems, University College Dublin, Dublin, Ireland; 4 Women in Global Health, Cairo, Egypt; 5 Duke Kunshan University, Kunshan, Jiangsu, China; 6 Medical Faculty, Department for Infectious Diseases and Tropical Medicine, Leipzig University, Leipzig, Germany; 7 National Ribat University, Khartoum, Sudan; 8 The Royal Melbourne Hospital, Melbourne, Victoria, Australia; 9 Highland Hospital, Department of Emergency Medicine, Alameda Health System, Oakland, California, USA; 10 Systems for Health Research Group, Swiss Tropical and Public Health Institute, Basel, Switzerland; 11 Institute of Development Studies, University of Sussex, Brighton, UK; 12 Faculty of Pharmaceutical Sciences and Technology, Chulalongkorn University, Bangkok, Thailand; 13 Department of Community Health, Tufts University, Medford, Massachusetts, USA; 14 NCD Alliance, Geneva, Switzerland; 15 Medstar, Georgetown University Hospital, Washington, District of Columbia, USA; 16 European Observatory for Health Systems and Policies, Brussels, Belgium

**Keywords:** Descriptive study, Health policy, Health systems, Public Health

## Abstract

**Background:**

While an estimated 70%–75% of the health workforce are women, this is not reflected in the leadership roles of most health organisations—including global decision-making bodies such as the World Health Assembly (WHA).

**Methods:**

We analysed gender representation in WHA delegations of Member States, Associate Members and Observers (country/territory), using data from 10 944 WHA delegations and 75 815 delegation members over 1948–2021. Delegates’ information was extracted from WHO documentation. Likely gender was inferred based on prefixes, pronouns and other gendered language. A gender-to-name algorithm was used as a last resort (4.6%). Time series of 5-year rolling averages of the percentage of women across WHO region, income group and delegate roles are presented. We estimated (%) change ±SE of inferred women delegation members at the WHA per year, and estimated years±SE until gender parity from 2010 to 2019 across regions, income groups, delegate roles and countries. Correlations with these measures were assessed with countries’ gender inequality index and two Worldwide Governance indicators.

**Results:**

While upwards trends could be observed in the percentage of women delegates over the past 74 years, men remained over-represented in most WHA delegations. Over 1948–2021, 82.9% of delegations were composed of a majority of men, and no WHA had more than 30% of women Chief Delegates (ranging from 0% to 30%). Wide variation in trends over time could be observed across different geographical regions, income groups and countries. Some countries may take over 100 years to reach gender parity in their WHA delegations, if current estimated trends continue.

**Conclusion:**

Despite commitments to gender equality in leadership, women remain gravely under-represented in global health governance. An intersectional approach to representation in global health governance, which prioritises equity in participation beyond gender, can enable transformative policymaking that fosters transparent, accountable and just health systems.

**WHAT IS ALREADY KNOWN ON THIS TOPIC**

- Women are historically under-represented in global health leadership positions, despite comprising over 70% of the workforce.
- Numerous commitments to gender equality in leadership, including global health governance, have been made at the international and multilateral levels (eg, the Beijing Platform).

**WHAT THIS STUDY ADDS**

- Between 1948 and 2021, 8% of the delegations to the annual World Health Assembly (WHA) have demonstrated gender parity (45%–55% women), while over 80% of delegations have been composed of a majority of men.
- Ever since the year 2000, only 13.5% of delegations displayed gender parity, with 69% (2887 out of 4186 delegations) still being composed of men majorities (2000–2021).
- Despite global commitments to gender equality in leadership, women remain under-represented in global health governance.
- Notably, not a single WHA in the past 74 years had more than 30% of the Chief Delegates as women (ranging from 0% to 30%).
- Based on our estimated trends, some countries may take over 100 years to reach gender parity (45%–55% women) in their WHA delegations and it is estimated to take over 40 years to achieve gender parity in the role of Chief Delegate across all WHA delegations.

**HOW THIS STUDY MIGHT AFFECT RESEARCH, PRACTICE OR POLICY**

- An intersectional approach to representation in global health governance, which prioritises equity in participation beyond gender, can enable transformative policymaking that fosters transparent, accountable, functional and just health systems.
- Urgent action is required by the global health community, with particular attention to regions and countries where progress has been stagnant.

## Introduction

Global and national health leadership continues to be dominated by men. While an estimated 70% of health workers are women, this percentage is not reflected in higher-wage healthcare occupations, nor the leadership roles of most international and national health organisations.^1^ This has continued to be illustrated during the COVID-19 pandemic; a study on gender representation in national COVID-19 task forces, revealed that only 3.5% of 115 identified COVID-19 decision-making and expert task forces had gender parity, and 85.2% were majority men.^2^ Additionally, while the economic contribution of women in global health is valued at US$3 trillion annually, half of women’s contribution is in the form of unpaid care work. The pay gap between men and women in healthcare remains around 28%.^1^ Women from low-income and middle-income countries are particularly under-represented in global health governance, holding less than 5% of senior leadership roles.^3^ Only 20% of global health institutions exhibit gender parity on their board of directors, and a mere 25% show gender parity at the senior management level. The current situation in global health leadership is reflected in broader national government positions. Over the last 5 years (as of April 2022), only 14% of countries had a woman as head of government, 21% as head of state and only 42% as Ministers of Health (*own analysis*).

Collective global efforts have led to greater advocacy for policies that have aimed to increase the status of women and girls—including their participation in political processes and governance. Yet, the overall under-representation of half of the world’s population in positions of leadership is still displayed in global decision-making bodies such as the World Health Assembly (WHA).^4^ The WHA is the central decision-making body of the WHO, the lead normative and technical actor within the global health sector, where priorities and agendas are set for the global community by delegations representing each Member State. In 2017 and 2018 WHA delegations reached a peak at 30% of Chief Delegates (head of delegation) being inferred as women. Yet in years to follow, progress has stalled, with merely 24% of delegations headed by a woman in 2019, 22% in 2020 and 24% in 2021 (*own analysis*).

Global health actors are increasingly aware of the unaddressed lack of gender diversity within global health governance. In 2020, the Gender Equal Health and Care Workforce Initiative was launched by WHO, the French government and Women in Global Health.^5 6^ As part of its commitments during the Generation Equality Forum in 2021, the WHO pledged to ‘promote and encourage gender parity in WHA delegations, WHO panels and advisory groups’.^5^

While progress to increase women’s representation in positions of leadership has been made across global health governance since the inception of the WHA in 1948, further action by Member States is likely needed to achieve gender equitable representation of WHA delegations. Here, we present a full analysis of gender representation of Member State, Associate Member and Observer (country/territory) WHA delegations over the past 74 years (1948–2021). This longitudinal, descriptive analysis serves to identify patterns of progress and/or stagnation across regions which can help orient priorities for action.

## Methods

### Data source and extraction

The official lists of delegates and other participants were obtained from the WHO’s Institutional Repository for Information Sharing (IRIS) (https://apps.who.int/iris/) for WHA1 (1948) through WHA74 (2021). In cases where multiple lists of delegates were available, the most recently dated list/document was used (the WHA resolutions and decisions document). A full list of the documentation used for each WHA can be found in online supplemental table 1.

10.1136/bmjgh-2022-009312.supp1 Supplementary data

For the purpose of this study, we primarily focused on delegations of Member States. We also included Associate Members, Observers for non-Member States (eg, Holy See, Order of Malta) and Observers with reference to specific resolutions (eg, Palestine (resolution WHA53.13), Chinese Taipei/Taiwan). While this predominantly included countries, it also included territories (eg, Tokelau, Puerto Rico), former political parties that acted as Observers in the earlier years of the WHA (eg, African National Congress, African Party for the Independence of Guinea and Cape Verde), representation of the Catholic Church (Holy See, Order of Malta) and de jure sovereign states (Palestine) that are under de facto control of another state. The scope of this research excluded other Observers such as representatives of international governmental organisations (IGOs) or non-governmental organisations (NGOs).

Country/territory, prefixes, (full) names, roles in the WHA (Chief Delegate, Deputy Chief Delegate, Delegate, Alternate, Adviser and other (eg, Secretary)), and occupational functions or affiliations (eg, Minister of Health, Secretary of State) were manually extracted and collected by 14 authors. Names were typically only provided with first initials rather than full names (eg, T. A. Ghebreyesus instead of Tedros Adhanom Ghebreyesus). A second member of the team double-checked all extracted data.

Data on whether the Head of State (HoS), Head of Government (HoG) and Minister of Health (MoH) of current Member States (2022) were inferred women in the past 5 years (2017–2022) were collected by five authors based on official governmental documentation/websites.^7^ This was double-checked by a second member of the team.

### Inferring likely gender

Likely gender was inferred based on gendered prefixes (eg, Ms., Mrs., Miss, Mr.) provided in the obtained lists of delegates (see online supplemental table 2). In case a gendered prefix was not available (eg, Dr, Prof.), gendered pronouns (eg, she/he/they) or other gendered language (eg, ‘husband’) used in WHO documentation or publicly available online documentation (eg, government websites, online biographies) was used to infer likely gender (eg, woman, man, non-binary) by 14 authors. If the full name was available or found through online searches, but no gendered prefixes, pronouns or other gendered language could be retrieved, a gender-to-name algorithm (https://genderize.io/) was used based on historical databases combining first name and country (n=3274, 4.6% of the inferred genders of delegation members). This tool has been applied and checked for robustness in multiple previous studies.^8–10^ The algorithm’s inferred gender was only accepted when the probabilistic certainty score was ≥0.50. If a likely gender could not be inferred after this approach, it was classified as ‘unknown’ (n=4383, 5.8% of total delegation members). Due to the inability of gender-to-name algorithms to identify people outside the gender binary and their reduced quality for inferring gender for non-Western names, this option functioned as a last resort. Gender was not inferred based on gender expression/presentation (phenotype) in images/photos from delegation members, due to the subjectivity of this method. As few people were inferred to be non-binary (n=2, 0.003% of delegation members), they were included as a gender minority in the categorisation of ‘women’ for the purpose of this analysis.

### Data cleaning and coding

We assigned current Member States to their corresponding WHO region group (2022) (Africa, Americas, Eastern Mediterranean, Europe, South-East Asia, Western Pacific),^11^ United Nations (UN) region group (2022) (Africa, Asia and Pacific, Eastern Europe, Latin America and Caribbean, Western Europe and Others),^12^ World Bank (WB) region group (2022) (East Asia and Pacific, Europe and Central Asia, Latin America and Caribbean, Middle East and North Africa, North America, South Asia, sub-Saharan Africa),^13^ WB income group (2022) (high-income, upper-middle-income, lower-middle-income, low-income)^13^ and 2019 Gender Inequality Index (GII) (see online supplemental table 14).^14^ Of the three different geographical regions (WHO, UN and WB), we focus on the WHO grouping in the main text—while results by UN and WB geographical groupings are provided in the supplement for additional context/information. The United Nations Development Programme GII measures gender inequalities in three aspects of human development: health, empowerment and economic status. GII ranges from 0 to 1, with higher GII values corresponding to increased disparities between women and men.^14^ Data from two of the Worldwide Governance Indicators (2019) were also obtained; the Voice and Accountability indicator (*‘*a reflection of the perceived extent to which a country’s citizens are able to participate in selecting their government, freedom of expression, freedom of association and free media^15 16^’) and Government Effectiveness indicator (*‘*a reflection of the perceived public services quality, civil service quality and degree of independence from political pressure, policy formulation and implementation quality, and the credibility of government’s commitment to policies^15 16^’). Estimates of governance performance on these indicators ranges from 2.5 (strong) to −2.5 (weak).^15 16^

Countries that changed their name (but not geographical boundaries) over the past 74 years have been re-coded to their current (2022) name (eg, Swaziland to Eswatini, Burma to Myanmar) to enable longitudinal analysis. Countries that have changed their geographical boundaries and/or geopolitical context have not been re-coded (eg, Yugoslavia, Ruanda-Urundi). Online supplemental table 3 displays the re-coded and not re-coded (former) countries, territories and political parties, including their relevant geopolitical contexts. To include these (former) countries, territories and political parties in regional longitudinal analyses, we grouped them under the geographical ‘UN’, ‘WHO’ and ‘WB’ regions that they would theoretically fall in based on their geographical location (eg, Yugoslavia was categorised as ‘Europe’, ‘Eastern Europe’ and ‘Europe and Central Asia’ for the ‘WHO’, ‘UN’ and ‘WB’ regions, respectively) as seen in online supplemental table 4. The WB income group and GII were not extended, as this would have required longitudinal data reflecting the countries change in income group and GII over 1948–2021.

### Data analysis and visualisation

We present backward 5-year rolling averages for the percentage (%) of women across WHO, UN, WB region and income groups and across delegates’ roles (eg, Chief Delegate) to generate time series over 1948–2021. Inferred gender composition of each delegation was further categorised into majority women (>55% women), gender parity (45%–55%) and majority men (>55% men)—this was presented as total number of delegations and percentage of delegations with majority women, gender parity and majority men over time (1948–2021).

Binomial 95% CIs were calculated for the proportions of interest (per cent of women). To estimate the per cent of change in women per year ±SE, we first aggregated the data over intervals of 10 years (backwards), and calculated the overall per cent of women over these intervals. This was then used to fit a linear regression model and to estimate the number of years until gender parity with 2010–2019 as baseline. The (i) estimated proportion ±95% CI of inferred women delegation members at the WHA in 2019, (ii) estimated change (%)±SE of inferred women delegation members at the WHA per year and (iii) estimated years±SE until gender parity from 2010 to 2019—were presented by WHO region, income group, WHA function and country. The p values for trend (β) were adjusted using the false discovery rate. The former (i, ii and iii) were presented separately for countries that were Member States in 2019 with an adjusted p value for trend <0.01, 0.01>p value<0.05 and p value>0.05. This (i, ii and iii) was separately presented for the three Observers in 2019 (Order of Malta, Holy See and Palestine).

Selecting only the countries with an adjusted p value for trend (β) of <0.05—i, ii and iii were plotted at a country level against the GII (2019), the Voice and Accountability Worldwide Governance Indicator (2019) and the Government Effectiveness Worldwide Governance Indicator (2019). Linear regression models were fitted and the Pearson’s Correlation Coefficient was calculated. To assess whether there is a difference in the distribution of i, ii and iii between countries who have had a woman HoS, HoG or MoH in the past 5 years (2017–2022) and those who have not—the non-parametric Wilcoxon signed-rank test was used, and distributions were presented using boxplot violin plots.

Missing values were excluded from all analyses. All statistical analyses and data visualisations were conducted in Stata V.16 and R V.4.0.5 (R Foundation, Vienna, Austria, www.r-project.org). For data visualisation, the tidyverse, dplyr, pals, and ggplot packages were used.

### Ethical considerations

All data used for this study were not restricted nor sensitive, nor did they require permission to access or collate. Data were publicly available and accessible, eliminating the need for additional ethical approval.

### Research team

The research team was composed of an internationally diverse group of researchers from a wide variety of socio-cultural backgrounds and languages (Arabic, Bengali, Chinese (Mandarin), Dutch, English, Farsi, French, German, Hausa, Kazakh, Nepali, Polish, Russian, Spanish, Swahili, Urdu, Yoruba) which allowed the team to include non-English/non-Western sources and perspectives.

## Results

A total of 75 815 delegates, representing 10 944 delegations of 228 unique (including former) countries, territories and political parties of Member States, Associate Members and Observers were included over 1948–2021. Online supplemental table 6 exhibits summary characteristics of all collected data.

**Figure 1:**
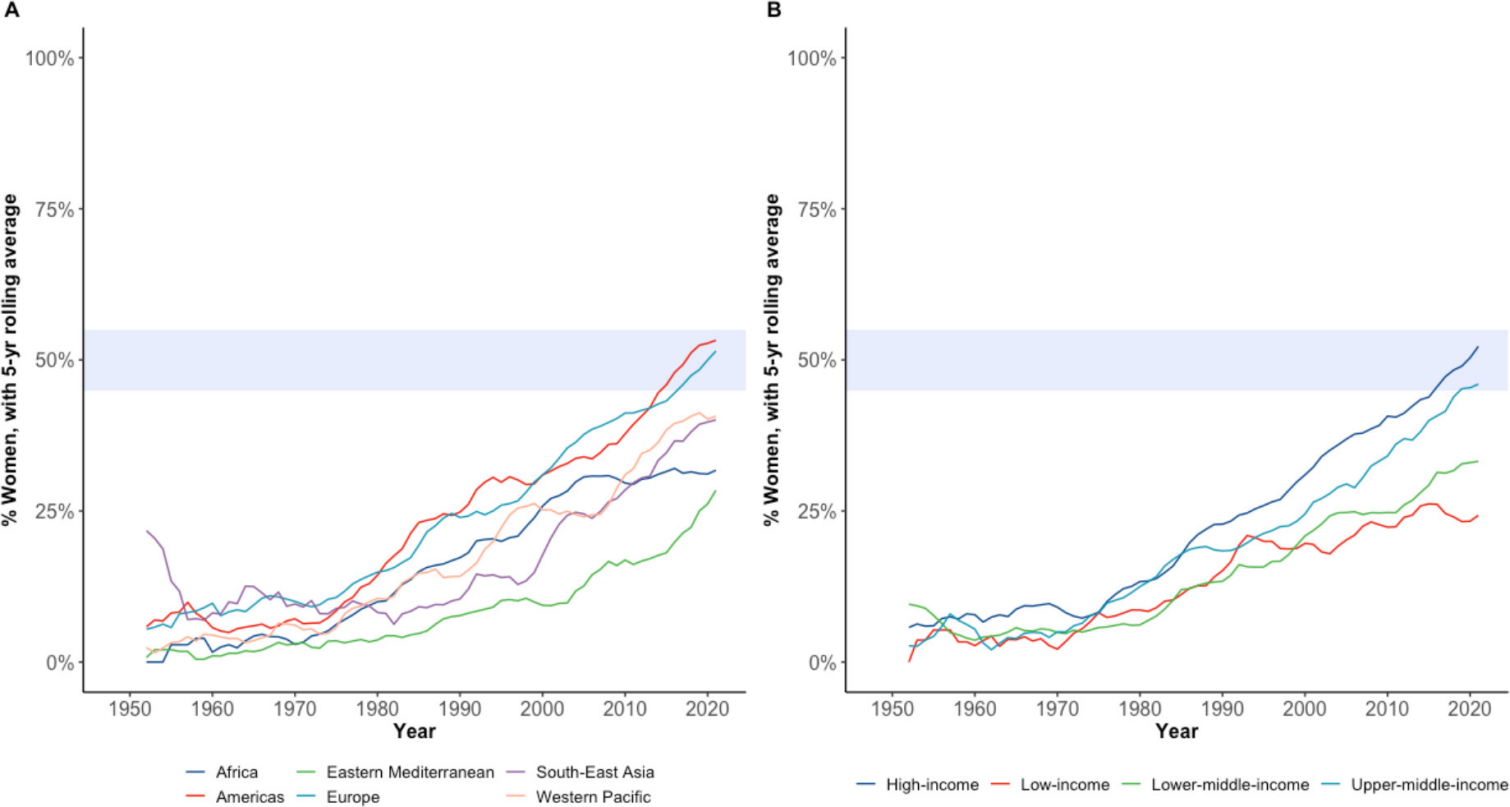
Proportion (%) of inferred women delegation members at the World Health Assembly over the years (1948–2021) by (A) WHO region, and (B) country income group (World Bank).

**Figure 2:**
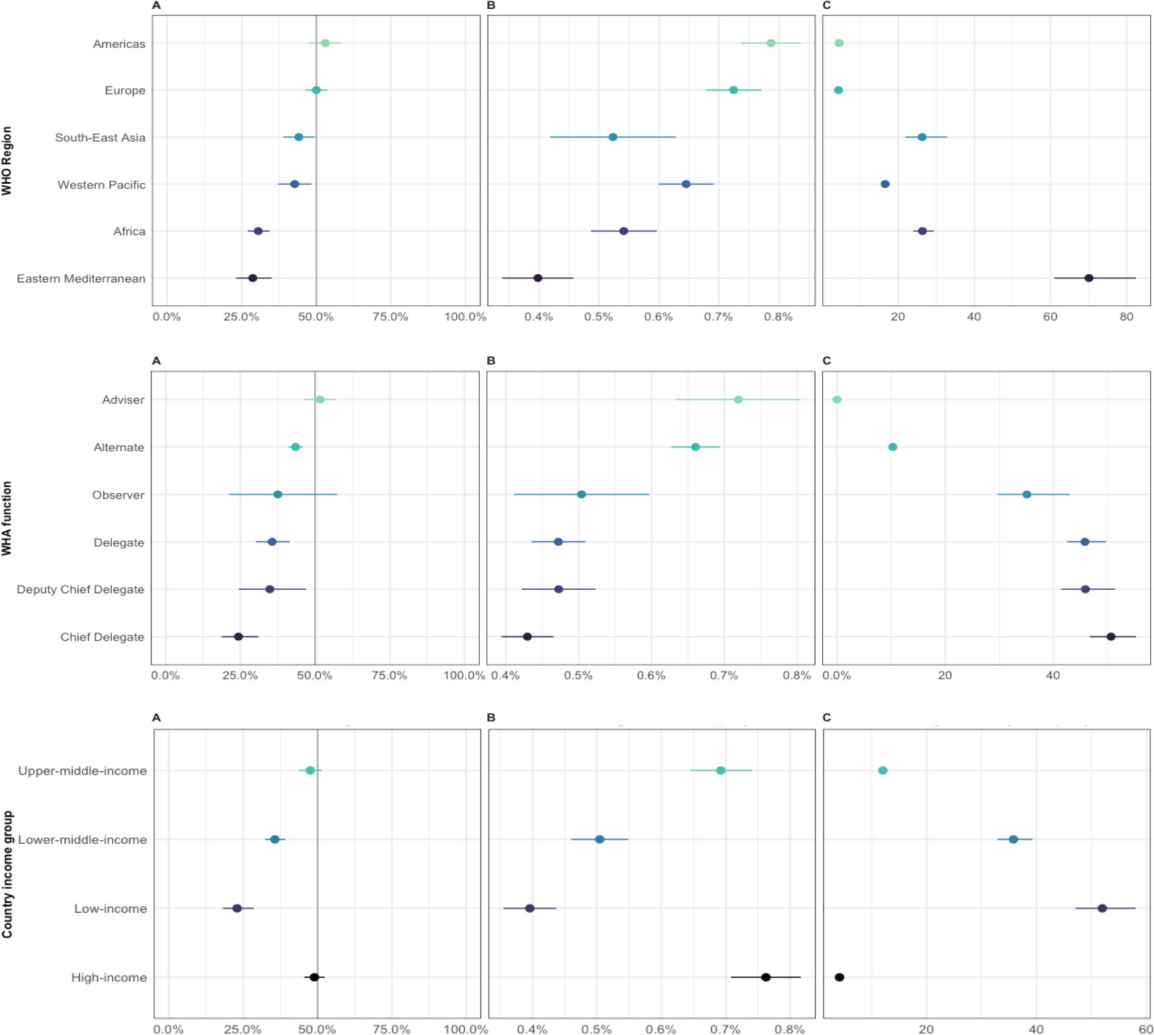
Women’s representation at the World Health Assembly (WHA) by WHO region (top), WHA function (middle) and country income group (bottom). (A) Proportion (%)±95% CI of inferred women delegation members at the WHA in 2019. (B) Estimated change (%)±SE of inferred women delegation members at the WHA per year. (C) Estimated years±SE until gender parity (45%–55% inferred women) from 2010 to 2019.

Overall, upward trends could be observed in the percentage of inferred women delegates over 1948–2021 across different WHO regions and income groups (figure 1). The Americas and Europe have seen gender parity achieved in their delegations within the last decade. Simultaneously, the Eastern Mediterranean Region, despite significant progress over time, has women representing just 25% of the WHA delegations—while the African region has had stagnant representation of women of around 25%–30% over the last 20 years (figure 1A). Similar trends can be observed when using different regional groupings, such as the UN and WB regional groupings (online supplemental figure 1). Based on current trends in the per cent increase of women’s representation per year, some WHO regions will take at least several decades before reaching gender parity of WHA delegation members across their region (figure 2, top). When assessing women’s representation across WB income groups, a widening gap of women’s representation between high-income and low-income countries in recent decades can be observed. Representation of women in low-income country delegations has been stagnant around 20%–25% since the 1990s, whereas women’s participation in overall high-income country delegations has doubled in that time, reaching gender parity in that income group (figure 1B). Based on current trends, it is estimated that the overall low-income country group will take around 50 years to achieve gender parity (figure 2, bottom).

**Figure 3:**
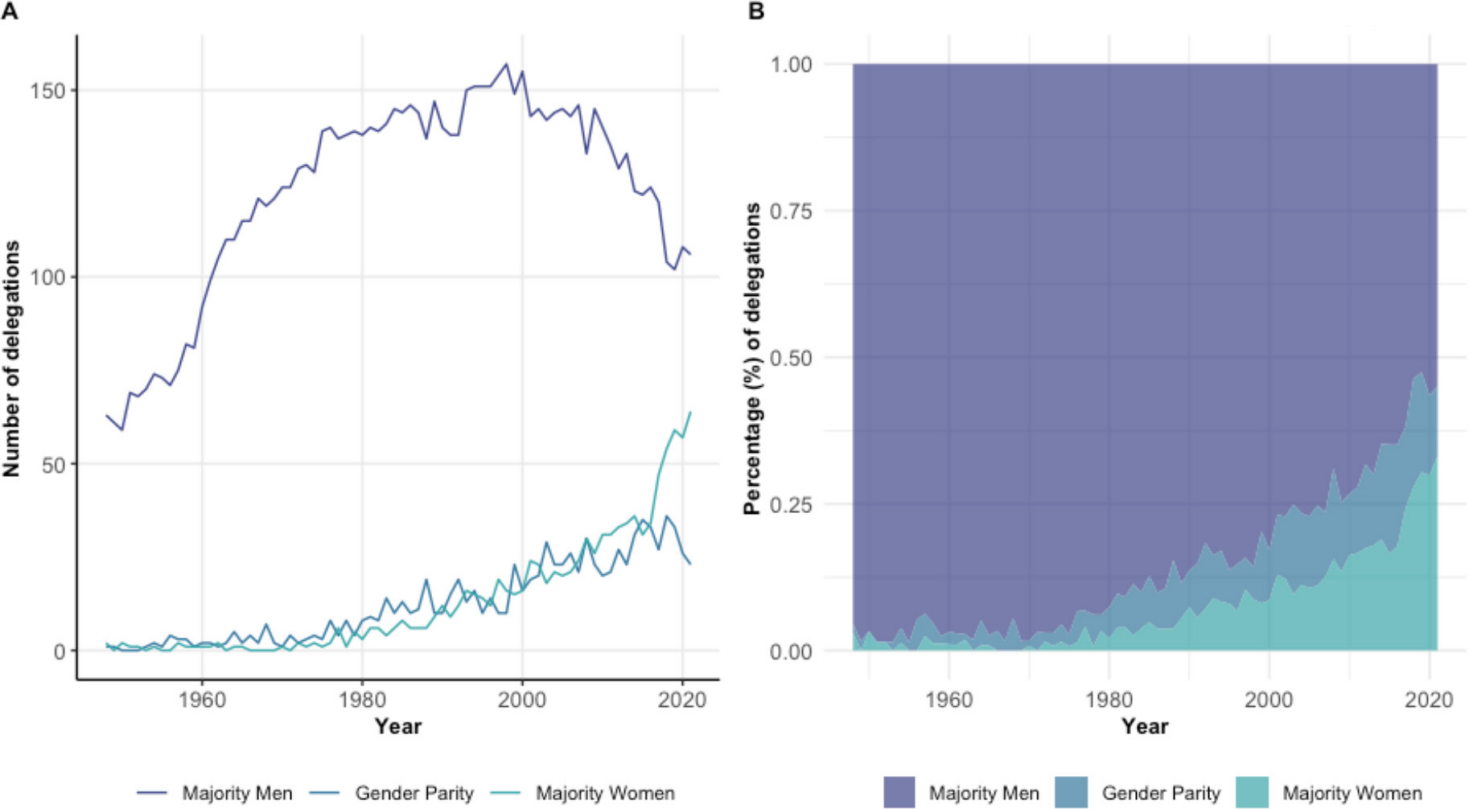
Inferred delegation composition at the WHA over the years (1948–2021). Majority men (>55% inferred men), gender parity (45%–55% inferred women) and majority women (>55% inferred women). (A) Number of delegations with majority men, gender parity or majority women. (B) Proportion of delegations with majority men, gender parity or majority women.

Across the entire time period of 1948–2021, 82.9% of delegations (n=9068 of 10 944 delegations) had a majority of men representing their delegation, 8.9% a majority of women (n=972) and 8.3% demonstrated gender parity (n=904). General trends in the composition of delegations can be observed over 1948–2021 with a decreasing number of majority men delegations and increasing number of delegations with gender parity or majority women (figure 3). Yet, even after 2000, only 13.5% of all WHA delegations displayed gender parity, with 69% (2887 out of 4186 delegations) still being composed of majority men (2000–2021). In 2021 (WHA74), it was estimated that 54.9% of delegations (106 out of 192) were majority men, 22.1% majority women and 23% displayed gender parity. While the COVID-19 pandemic impacted the total number of WHA delegates in 2020, 1655 delegates in 2020 versus 2583 delegates in 2019 (online supplemental figure 2), the percentage of total inferred women was 42% in both 2019 and 2020. In 2021, delegation size and percentage of women delegates rose to 46% inferred women of 2274 total delegates.

Women have been less represented in higher-powered delegation roles (here considered to be Chief Delegate and Deputy Chief Delegate) in all WHO regions over 1948–2021. Less than 30% of Chief Delegates and Deputy Chief Delegates were inferred to be women in 2021, ranging from 0% to 30% (see online supplemental figure 3). At the current rate of change, it is estimated to take over 40 years to achieve gender parity in the role of Chief Delegate across all WHA delegations (figure 2, middle). In contrast, women’s participation in WHA delegations is higher in the roles of an Adviser or Alternate, with over 55% of delegation advisers being women in 2021 (online supplemental figure 3A).

**Figure 4:**
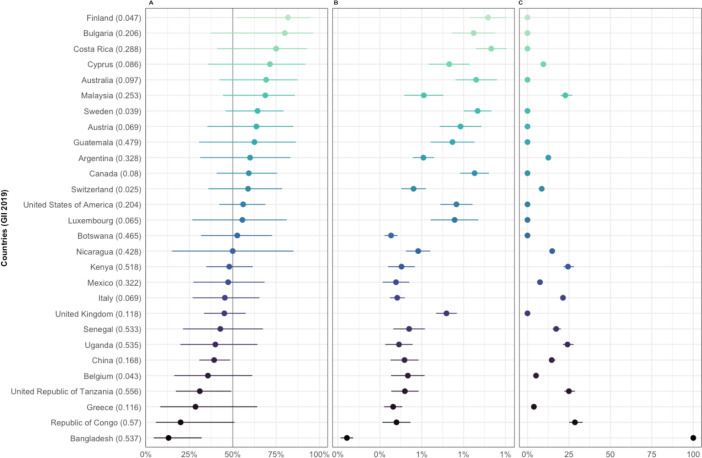
Women’s representation in countries with a trend in estimated change of percentage women delegation members per year adjusted p value<0.01. (A) Proportion (%)±95% CI of inferred women delegation members at the World Health Assembly in 2019. (B) Estimated change (%)±SE of inferred women delegation members at the World Health Assembly per year. (C) Estimated years±SE until gender parity (45%–55% inferred women) from 2010 to 2019. Note, only countries that were Member States in 2019 are included. GII, Gender Inequality Index.

**Figure 5:**
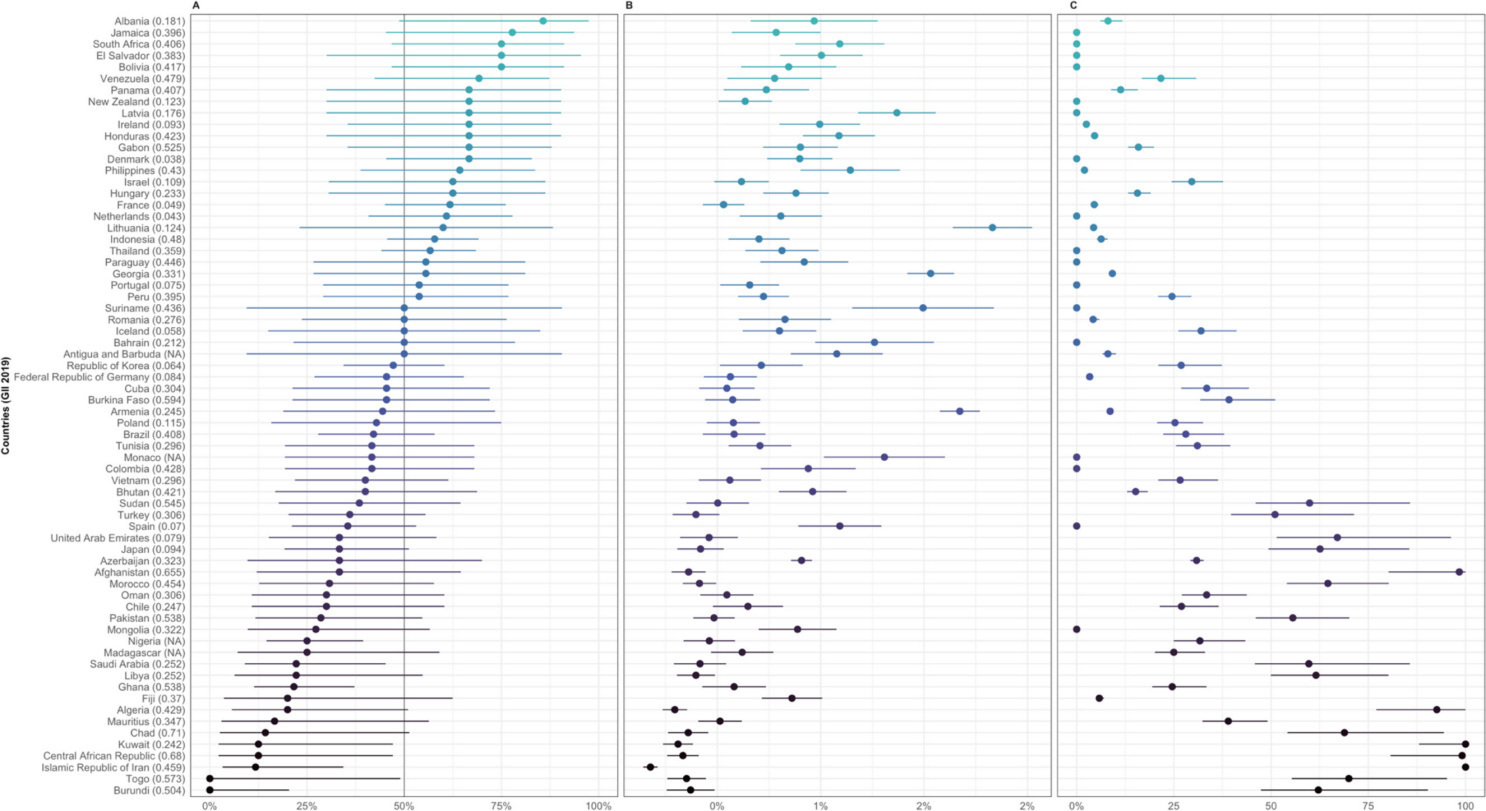
Women’s representation in countries with a trend in estimated change of percentage women delegation members per year adjusted p value<0.05 but>0.01. (A) Proportion (%)±95% CI of inferred women delegation members at the World Health Assembly in 2019. (B) Estimated change (%)±SE of inferred women delegation members at the World Health Assembly per year. (C) Estimated years±SE until gender parity (45%–55% inferred women) from 2010 to 2019. Note, only countries that were Member States in 2019 are included. GII, Gender Inequality Index.

Women’s representation in 2019 (i), per cent change in women’s representation by year (trend) (ii) and years until gender parity (iii) varies widely across Member States (figures 4 and 5 and online supplemental figure 4), and Observers (online supplemental figure 5). When comparing countries with adjusted p values<0.01 for trend (figure 4), only Bangladesh is estimated to take over 100 years to reach gender parity in their overall WHA delegation members from the 2010–2019 baseline—while others are expected to take several years (eg, Mexico, Greece, Uganda), or have already reached gender parity (eg, Finland, Argentina). More variation can be observed when comparing the countries with adjusted p values for trend of >0.01 and <0.05 (figure 5)—with several countries estimated to take at least several decades before reaching gender parity in their WHA delegations. While, i, ii and iii are also presented in online supplemental figure 4 for Member States with adjusted trend p values>0.05, interpretation of ii and iii should be done with caution.

**Figure 6:**
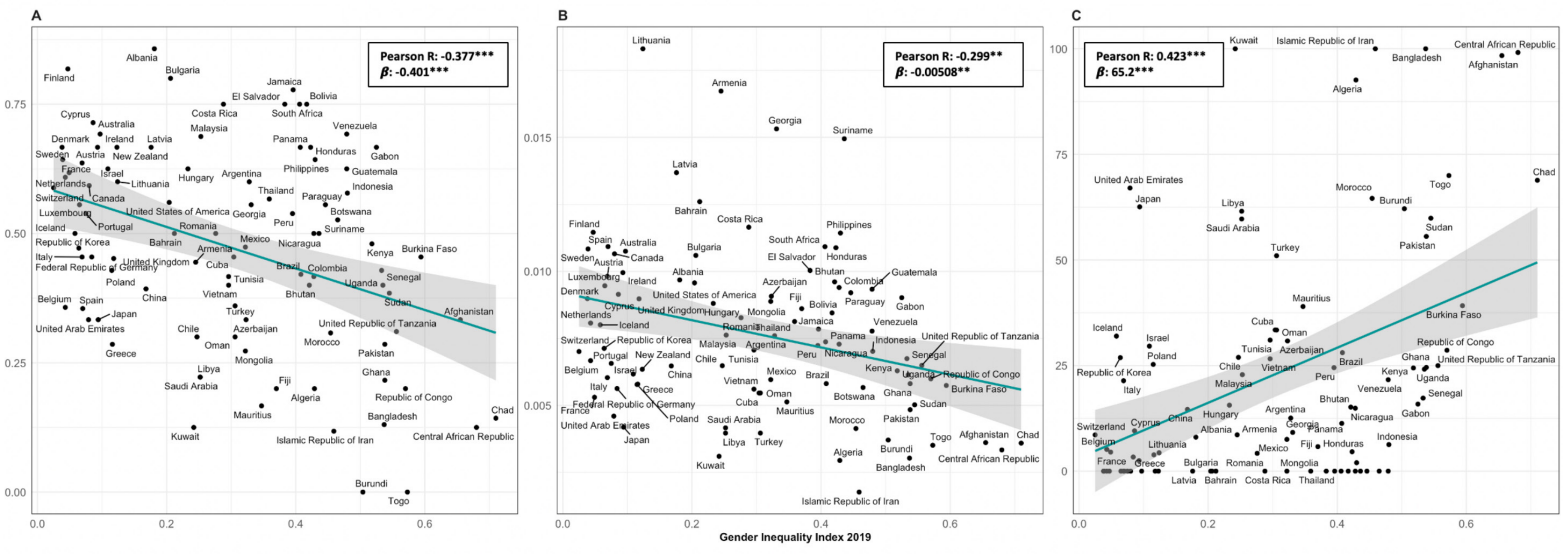
Women’s representations by Member State’s Gender Inequality Index (GII) 2019. (A) Proportion (%)±95% CI of inferred women delegation members at the World Health Assembly in 2019 by GII 2019. (B) Estimated change (%)±SE of inferred women delegation members at the World Health Assembly per year by GII 2019. (C) Estimated years±SE until gender parity (45%–55% inferred women) from 2010 to 2019 by GII 2019. Note, only countries with a trend in estimated change of percentage women delegation members per year adjusted p value<0.05 were included.

When plotting i, ii and iii against the GII at a country level, statistically significant negative trends with the per cent of women delegates in 2019 (figure 6A) and the per cent change in women delegates per year can be observed (figure 6B); statistically significant positive trends are observed with the number of years until parity and GII (figure 6C). In other words, a higher GII seems to be correlated to lower women’s representation in WHA delegations (figure 6). Similarly, when plotting i, ii and iii against the *Voice & Accountability* and *Government Effectiveness* Worldwide Governance indicators, statistically significant positive trends can be observed with the per cent of women delegates in 2019 (online supplemental figure 6A, 7A) and per cent change in women delegates per year (online supplemental figure 6B, 7B), while significantly negative trends can be observed with years until parity (online supplemental figure 6C, 7C). This may suggest correlations between women’s representation and governance performance on these indicators.

Lastly, when comparing the distribution of i, ii and iii, no significant differences in the distributions were observed between countries with a woman HoS or HoG and countries without a woman HoS or HoG in the past 5 years (2017–2022). However, statistically significant differences could be observed for the distributions of i, ii and iii between countries that had a woman MoH in the past 5 years—with a higher per cent of women being part of 2019 delegations in countries who had a woman MoH (p value=0.004), a higher per cent change in women delegation members per year (p value=0.003) and a lower number of years until parity (p value=0.009) compared with countries who did not have a woman MoH.

## Discussion

While highlighting the progress of women’s representation in WHA delegations over time, this study presents a timely expose on the prevailing levels of gender inequality and exclusion of gender-diverse voices in global health leadership and decision-making bodies. Our quantitative data from 10 994 delegations and 75 815 delegation members spanning 74 years (1948–2021), illustrates that men remain over-represented in most WHA delegations of Member States, Associate Members and Observers to date. From 1948 to 2021, 82.9% of delegations were represented by a majority of men and no WHA had more than 30% of women Chief Delegates in the 74-year period. Wide variation in trends over time could be observed across different geographical regions, income groups and countries. This is likely the result of different prolonged and multifaceted context-specific social, cultural and institutional factors that inhibit meaningful equitable participation within different countries. Based on our estimated trends, some countries may take over 100 years to reach gender parity (45%–55% women) in their WHA delegations. The lack of representation at this lead global health governance platform is in stark contrast to the health workforce where women constitute over 70%. This unjust disparity in representation fuels real world inequities experienced by women globally.

Unsurprisingly, a higher GII, which indicated higher disparities between women and men in a country, seems to be correlated with a lower proportion of women delegation members in 2019, lower per cent change per year and more years until gender parity. Simultaneously, correlations with the aggregate WB’s *Voice & Accountability* and *Governance Effectiveness* indicators seem to have the opposite direction. Countries that are perceived to have a strong performance on the *Voice & Accountability* indicator (citizen’s participation in selecting their government, freedom of expression, association and free media) as well as countries that have a perceived strong performance on the *Governance Effectiveness* indicator (quality of public and civil services, quality of policy formulation and implementation and credibility of government’s commitments to such policies) are estimated to have a higher proportion of women delegation members, higher per cent change per year and fewer years until gender parity. These indicators may serve as a proxy for other factors related to the political and socioeconomic context, history and culture of a country that influence its societal norms and structures which may enable higher gender equality and/or participatory governance.^17^ Importantly, while useful for broad cross-country comparisons and trends over time, these broad composite represent complex phenomena which cannot be used to elucidate direct or clear associations and therefore to inform specific action for governance reforms.^15^ Hence interpretation of these correlations should be approached with caution.

The progress seen today may partially be attributed to a culmination of decades of advocacy, focused on gender equality in international governance. Established in 1946, UN Commission on the Status of Women was the first global intergovernmental body within the UN entirely dedicated for advocating gender equality and the empowerment of women.^18^ Nearly 50 years later, the 1995 Beijing Platform for Action was adopted at the World Conference on Women in Beijing, highlighting ‘Women in power and decision-making,’ as one of the 12 critical areas where urgent action was demanded to ensure greater equality for women and girls.^19^ In the Beijing Platform for Action, 189 country governments committed to having women in 30% of their decision-making roles and the proportion of women in countries’ governing bodies nearly doubled since.^20^ Subsequent years continued to witness a growth of women in leadership roles across global health governance, including Dr Gro Harlem Brundtland, who served as the first woman in the role of WHO Director-General in 1998.^21^ These changes emerging from global governance platforms and international declarations/commitments, are accompanied by overall societal shifts surrounding gender equity such as changing perspectives around gender roles, identities and expectations—enabling improvements in women’s participation in governance and leadership across the world.^22^

### Policy implications: responsibilities of the WHO

Through its leadership and normative authority, the WHO holds a central role in promoting gender equity in global health leadership. Global health governance benefits from the inclusion of a variety of perspectives in order to inform more comprehensive and transformative health systems programmes and policies.^2 23 24^ Diverse teams (gender, ethnicity, etc) tend to be higher performing, are more innovative and can contribute to inclusion and equality in wider communities.^25^ Role-modelling diversity in WHO staff (not only in gender, but also across other socio-demographic factors through an intersectional lens) encourages other global health organisations and governments to follow suit, and use the rich dividends of diverse expertise, experiences and perspectives in global health.

WHO has signalled its commitment to promoting gender equity in WHA delegations,^5^ and could consider more active strategies to ensure this is achieved. This may include supporting Member States to develop leadership programmes or the implementation of gender diversity quotas within delegations. However, it is important to recognise that while gender quotas can be an important method to establish standards for representation, they do not directly correlate to an influence in decision-making. Furthermore, an increased number of women at the table does always or necessarily equate to more gender-diverse, inclusive and improved decision-making. For example, women may still not have as many opportunities to speak, access to power and quotas do not take into consideration the formal and informal mechanisms through which gender inequity in leadership occurs.^26^ Policies on inclusive leadership should consider more than representation in numbers, but consider the entire enabling environment for the inclusion of diverse voices and perspectives using an intersectional approach to global health decision-making and policy.

The WHO could further commit to monitoring progress on delegation representation over time through the collection of data that is disaggregated by gender and other social identities that may affect the participation of under-represented people, perspectives and expertise in global health leadership.^27^ Tracking this information is an important factor for accountability—while presenting this information to Member States may also serve as powerful impetus for meaningful change. When collecting data on WHA participants, WHO should acknowledge that gender is not binary and provide appropriate options during registration, including the ability to self-identify as gender non-binary or -conforming.

### Policy implications: responsibilities of Member States

The responsibility for elevating this continuing imbalance in gender participation in WHA delegations lies with each individual Member State and should be supported by the WHO and the international community. While it is imperative to increase women’s participation in country delegations, women and gender minorities of diverse backgrounds and origin, should also be meaningfully included in leadership positions within government and international organisations. This would reflect their existing roles, work, expertise and contributions to the global health field, and will further inclusive engagement in conversations related to their own health and well-being. Advocates and academics alike have suggested a spectrum of interventions relating to environmental, institutional and individual factors to encourage and empower women’s continued involvement and leadership in global health roles.^28^ These structural and systematic interventions such as leadership grants, formal policies to safeguard women in the workplace and peer-training and mentorship opportunities could facilitate the meaningful participation in decision-making and leadership roles.^28 29^

Individual Member States play an important role in ensuring fair and equitable representation in global health governance, including in their WHA delegations. Member States with a current commitment to gender equity should remain dedicated to their individual country targets, as outlined by their recent and relevant commitments.^30 31^ Member States without a current commitment to gender equity in governance, could make explicit public commitments and adopt strategies, policies and practices to enable equitable participation in global health governance. As factors influencing equitable participation in global health governance will differ across settings, this will require active commitments to identify, assess and respond to the prolonged and multifaceted social, cultural and institutional factors that inhibit meaningful equitable participation in global health governance across different country contexts. A recent systematic review on leadership in health identified that cultural change and leadership commitment across five emergent categories were of particular importance to facilitate meaningful equitable participation in leadership: organisational processes, training and development, awareness and engagement, mentoring and networking, and organisational support tools.^32^

However, it is important to note that, the inclusion of more women does not explicitly assure the full spectrum of gender transformative policies nor can it be assumed that women are always gender-inclusive advocates.^26 33^ Beyond gender parity in representation, it is imperative to recognise that women are not a homogenous group and differences in class, income, race, religion, ability and nationality must also be considered in the development and implementation of global health policies.^26 27^ Tacking such an intersectional approach to global health can be used to address not only representation in global health multilateral systems, but the systemic inequalities and power hierarchies that influence power in global health decision-making.^2 24 26 27 34^

### Strengths and limitations

Our study has several strengths. The scope of this analysis provides the first comprehensive large-scale longitudinal quantitative assessment of delegation’s gender representation since the WHO’s inception in 1948. The data disaggregation enabled further descriptive evaluation of trends between countries, regions, income groups and delegate roles. Furthermore, the data generated provides a strong foundation for further gender equitable data collection and in-depth analysis the authors and/or WHO may want to commit to monitor progress in WHA participation over time.

However, our study also has several limitations. First, while our analysis enables the assessment of gender representation over time, it does not allow us to assess the influence delegates have on WHA decision-making processes—nor allow us to directly assess whether increasing gender diversity in WHA delegations may produce more equitable gender-transformative global health policies and agreements at the WHA. Arguably, many decisions on behalf of nations may have already been agreed on before the WHA actually convenes, limiting the influence of representative delegation members on formal decision-making processes.

Second, inferring likely gender was largely limited to binary definitions of gender, as authors were dependent on the prefixes and other gendered language used in WHO/online documentation and a binary gender-to-name algorithm, instead of delegates self-identification. As a result, some inferred genders may have misrepresented the gender identity of delegates. These limitations further point to the need for better data collection around gender and sex in order to promote transparency and accountability in gender-inclusive governance (eg, options for delegates to self-identify their gender in the WHA registration process).

## Conclusion

Despite some progress in recent decades, women continue to be under-represented in global health leadership and decision-making at the highest level. The ongoing under-representation of women has implications for not only gender equality but also for global health systems worldwide, from the global to local level. Prioritising equitable intersectional approaches, which prioritise equity of various forms beyond gender, and inclusive representation in decision-making enables transformative policy-making that fosters transparent, accountable, functional and just health systems. Urgent action is required by the global health community, with particular attention to regions and Member States (countries) where progress has been stagnant in the past 74 years.

## Data Availability

Data ara available in a public, open access repository. The data to support the findings of this study are available upon request from the corresponding author, and will soon be made publicly available on an online repository.
