# Supplementary material for: Does global health governance walk the talk? Gender representation in World Health Assemblies, 1948–2021
Source: BMJ Glob Health. 2022 Aug 23;7(8):e009312. doi: 10.1136/bmjgh-2022-009312 (PMC9403126; doi:10.1136/bmjgh-2022-009312)
Supplement: Supplementary data [file bmjgh-2022-009312supp001.pdf]

BMJ Publishing Group Limited (BMJ) disclaims all liability and responsibility arising from any reliance placed on this supplemental material which has been supplied by the author(s)

Does Global Health Governance Walk the Talk? Gender representation in World Health Assemblies over 1948-2021

Supplement Materials

van Daalen KR, *et al. BMJ Global Health* 2022; 7:e009312. doi: 10.1136/bmjgh-2022-009312

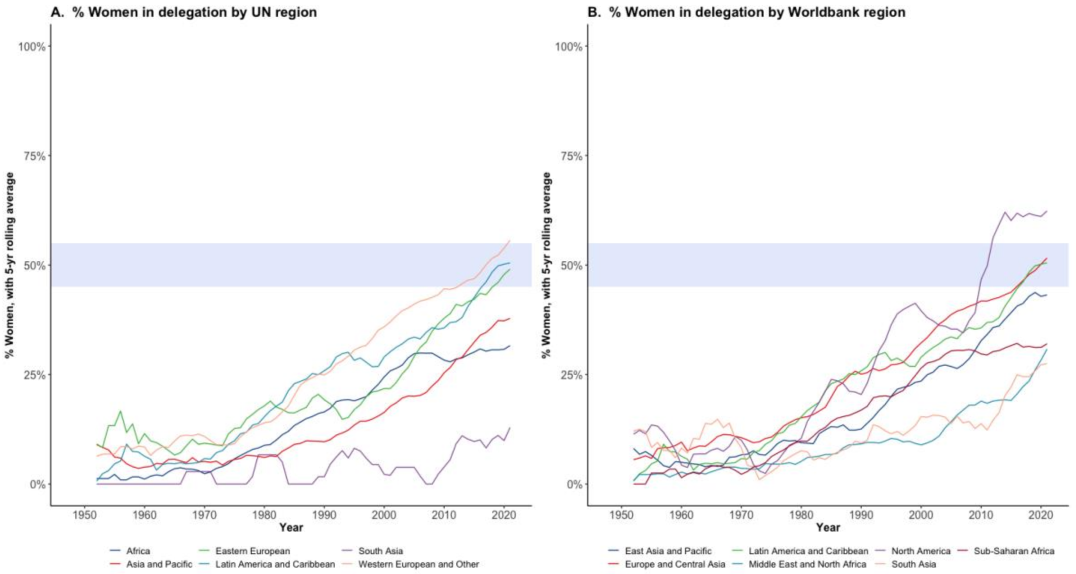

Supplement Figure 1. Proportion (%) of inferred women delegation members at the World Health Assembly over the years (1948-2021) by (a) UN region[1], and (b) World Bank region[2].

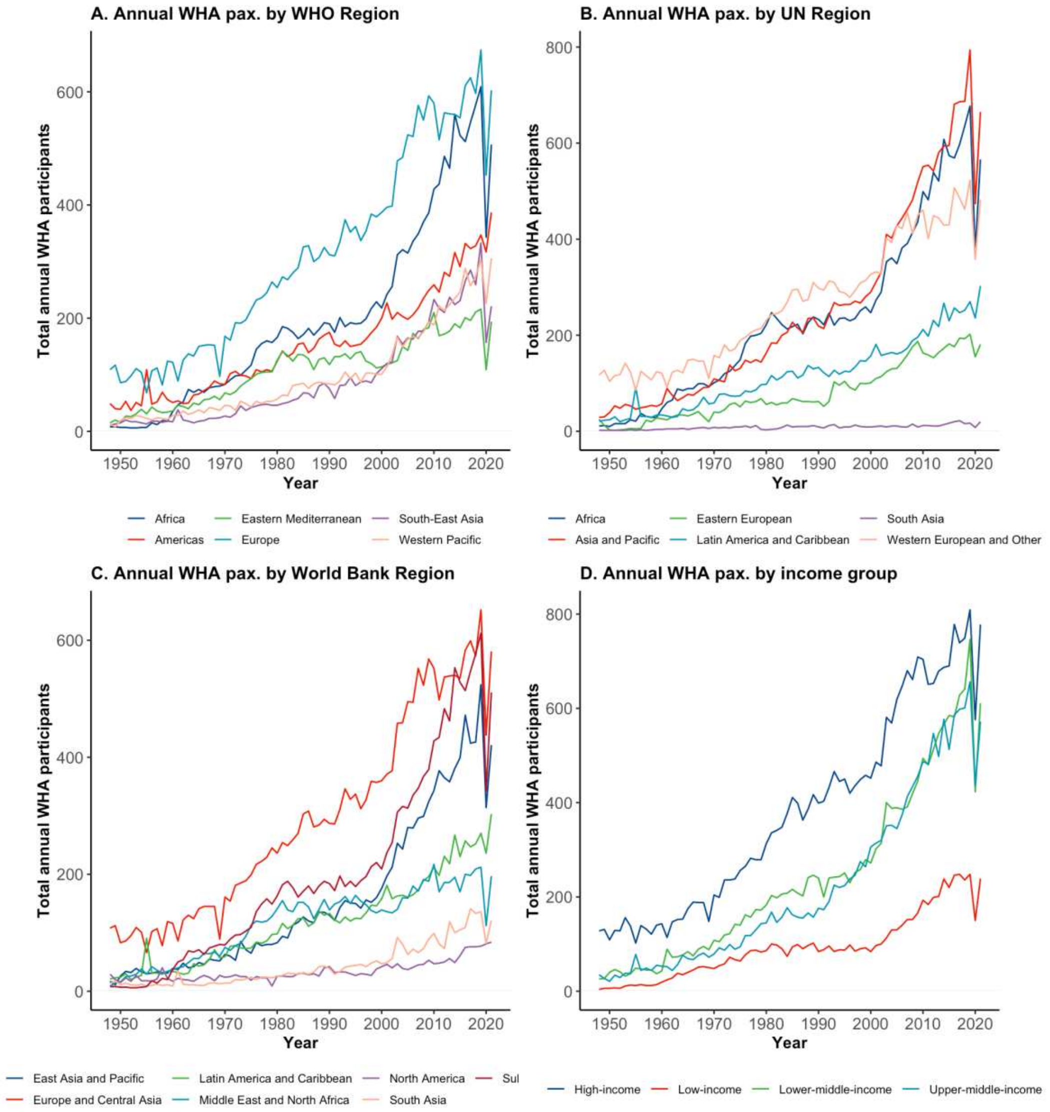

Supplement Figure 2. Total number of delegation members (pax.) at each World Health Assembly over the years (1948-2021). Member states, associate members and (permanent) observers are included. Groupings provided by (a) WHO region[3], (b) UN region[1], and (c) World Bank region[2] and (d) income group (World Bank)[2].

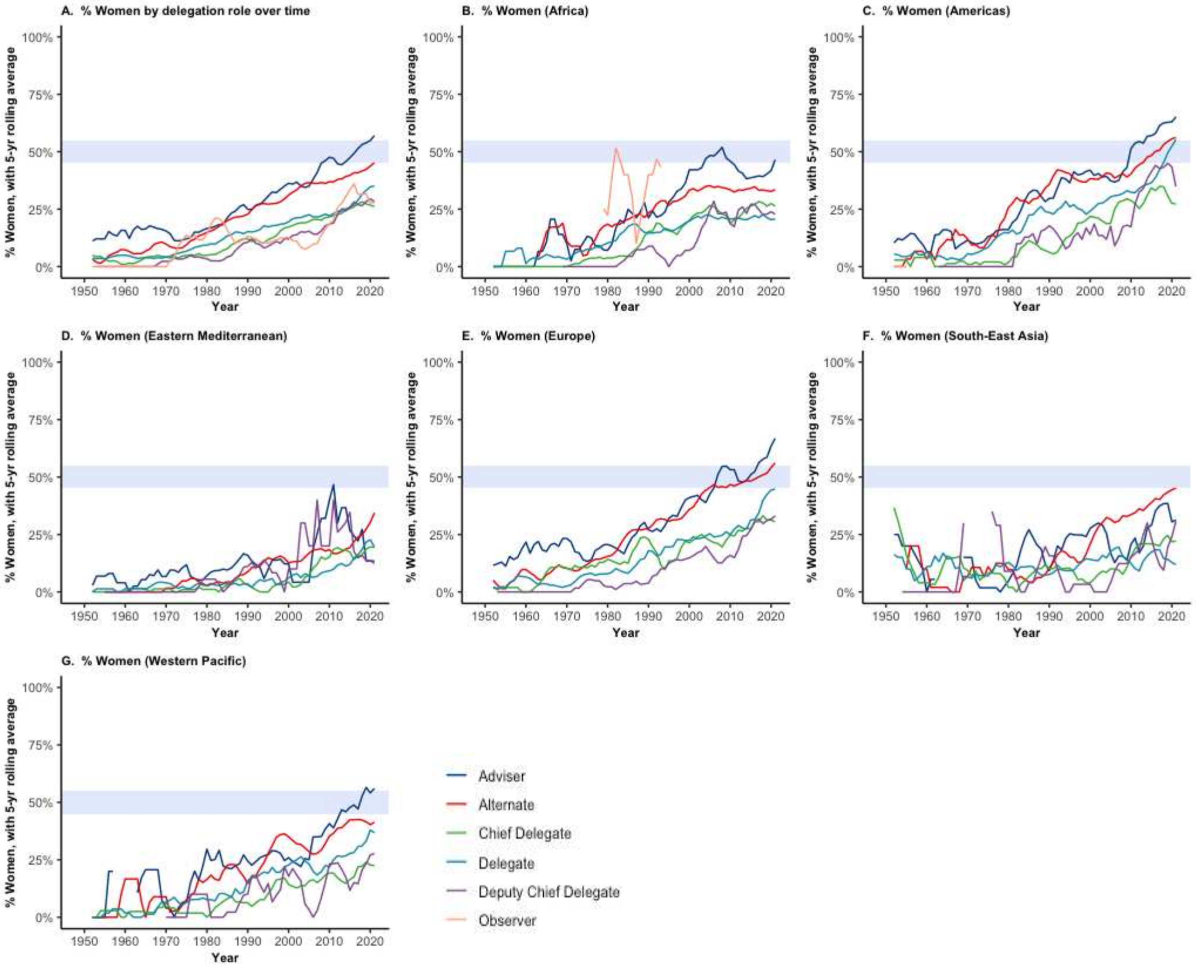

Supplement Figure 3. % Inferred women by delegation role over time (1948-2021) including (a) all delegates, and by WHO Region[3]; (b) Africa, (c) Americas, (d) Eastern Mediterranean, (e) Europe, (f) South-East Asia, (g) Western Pacific.

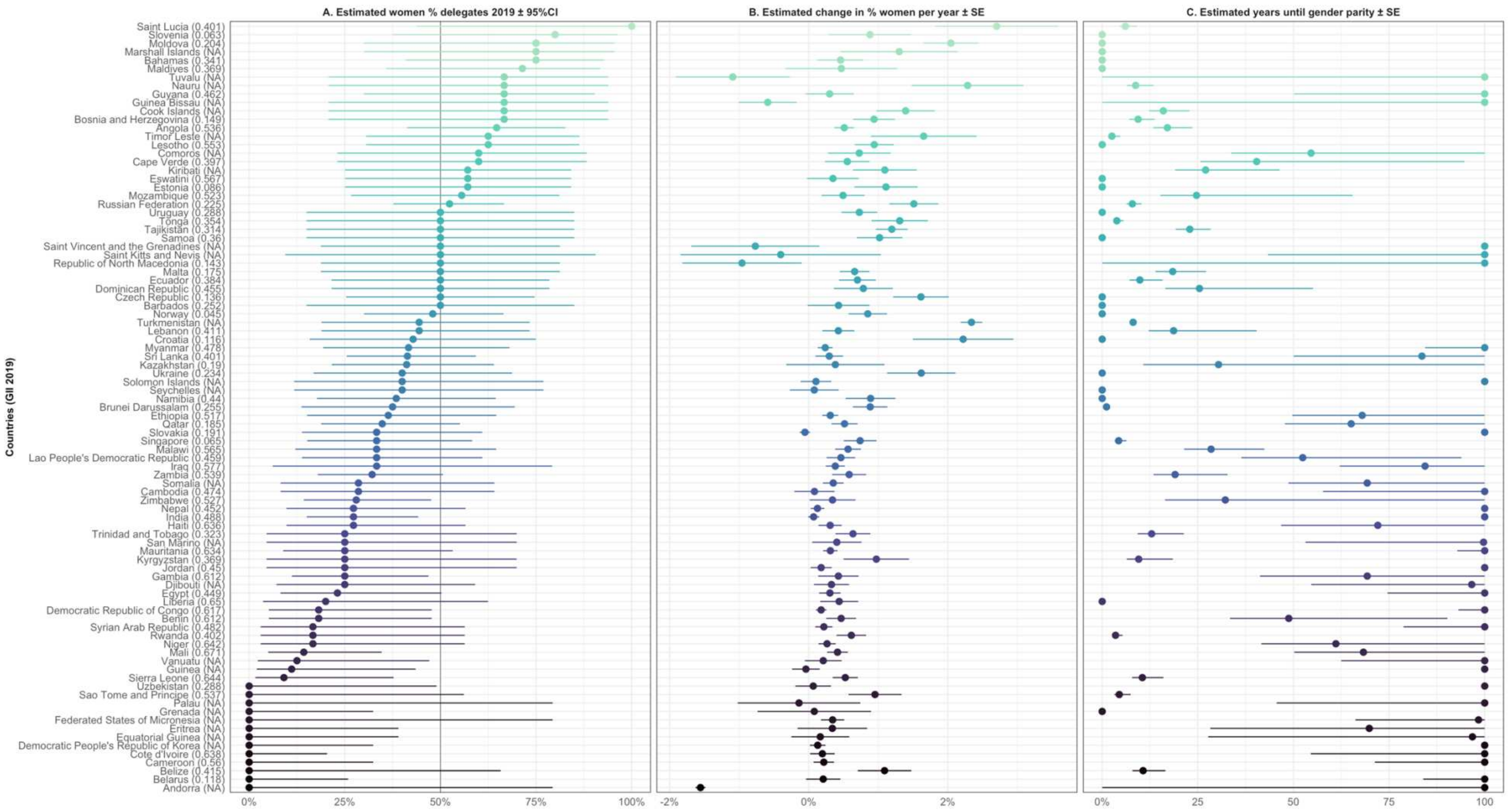

Supplement Figure 4. Women's representation in countries with trend in estimated change of % women delegation members per year adjusted *P*-value >0.05. (a) Proportion (%) ± 95% Confidence Interval (CI) of inferred women delegation members at the World Health Assembly in 2019. (b) Estimated change (%) ± standard error (SE) of inferred women delegation members at the World Health Assembly per year. (c) Estimated years ± standard error (SE) until gender parity (45%-55% inferred women) from 2010-2019. *Note, only countries that were Member States in 2019 are included.*

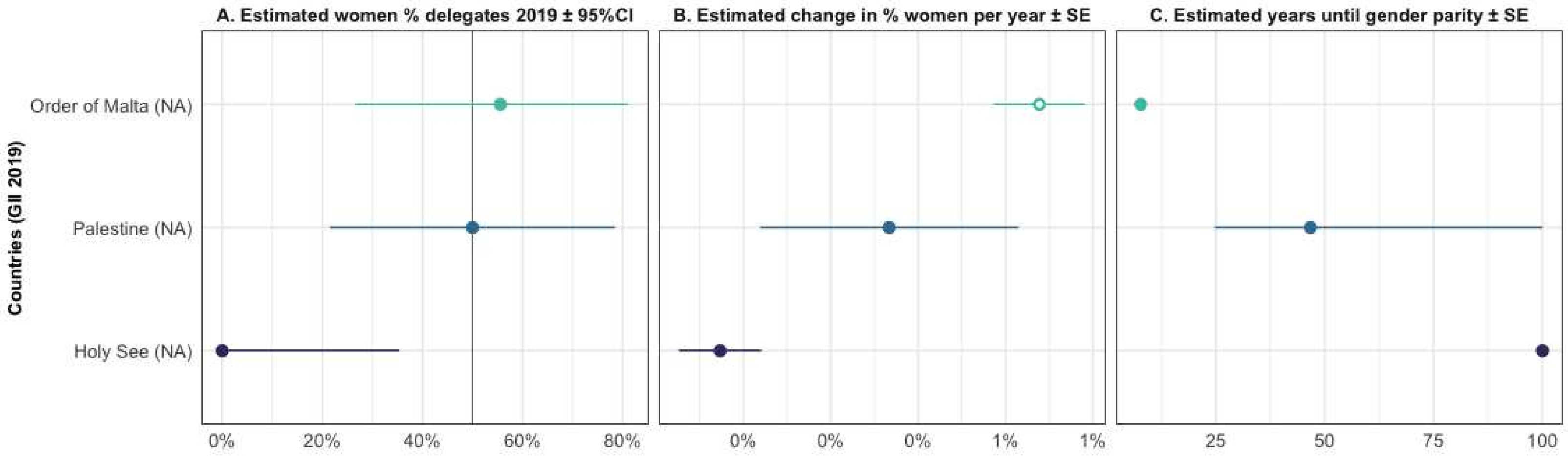

Supplement Figure 5. Women's representation in Observers of the 2019 World Health Assembly. White filled point: adjusted *P*-value for trend is <0.05. (a) Proportion (%) ± 95% Confidence Interval (CI) of inferred women delegation members at the World Health Assembly in 2019. (b) Estimated change (%) ± standard error (SE) of inferred women delegation members at the World Health Assembly per year. (c) Estimated years ± standard error (SE) until gender parity (45%-55% inferred women) from 2019.

van Daalen KR, *et al. BMJ Global Health* 2022; 7:e009312. doi: 10.1136/bmjgh-2022-009312

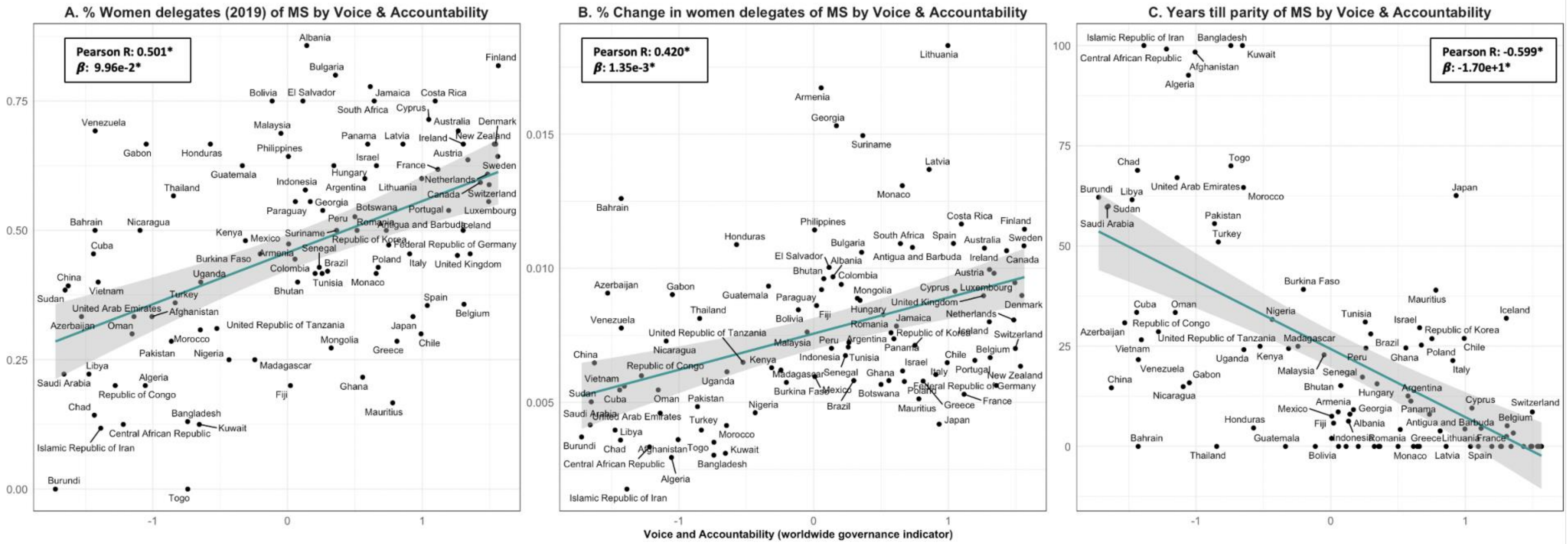

Supplement Figure 6. Women's representations by Member State's Voice & Accountability Worldwide Governance Indicator (2019). (a) Proportion (%) ± 95% Confidence Interval (CI) of inferred women delegation members at the World Health Assembly in 2019 by Voice & Accountability indicator 2019. (b) Estimated change (%) ± standard error (SE) of inferred women delegation members at the World Health Assembly per year by Voice & Accountability indicator 2019. (c) Estimated years ± standard error (SE) until gender parity (45%-55% inferred women) from 2010-2019 by Voice & Accountability indicator 2019. *Note, only countries with trend in estimated change of % women delegation members per year adjusted P-value <0.05 were included.* The Voice and Accountability Worldwide governance indicator is a reflection of the perceived extent to which a country's citizens are able to participate in selecting their government, freedom of expression, freedom of association and free media. Estimates of governance performance on these indicators ranges from 2.5 (strong) to -2.5 (weak).[4,5]

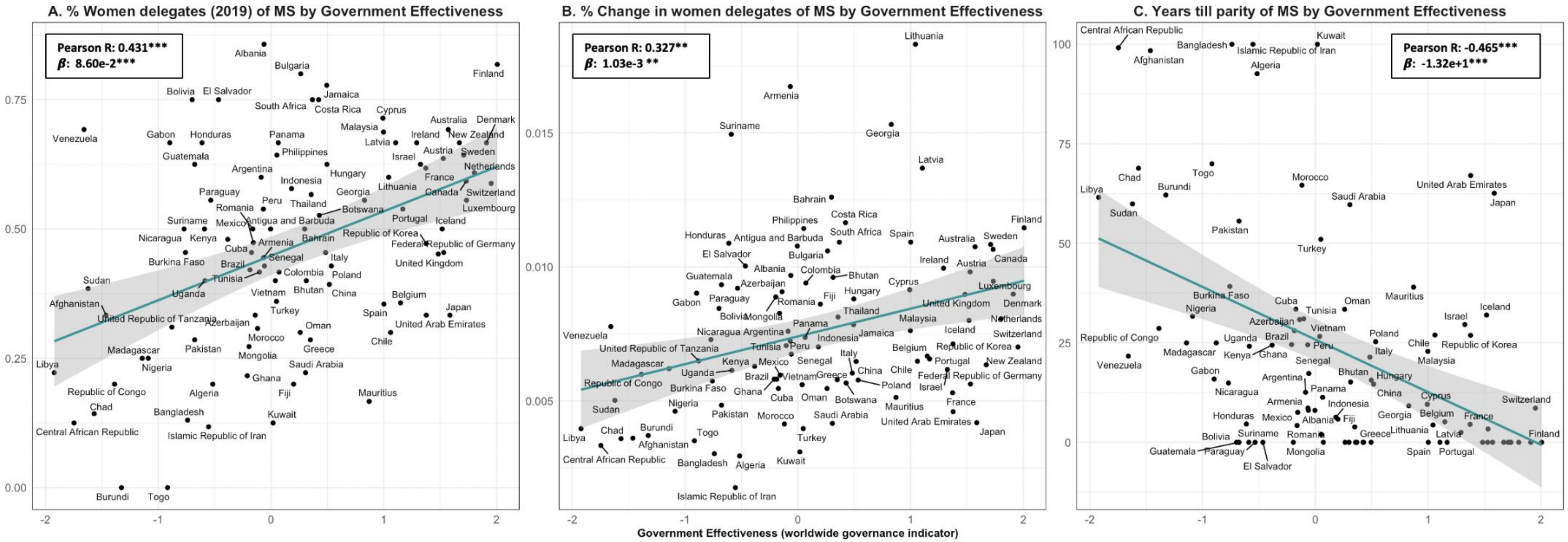

Supplement Figure 7. Women's representations by Member State's Government Effectiveness Worldwide Governance Indicator (2019). (a) Proportion (%) ± 95% Confidence Interval (CI) of inferred women delegation members at the World Health Assembly in 2019 by Government Effectiveness indicator 2019. (b) Estimated change (%) ± standard error (SE) of inferred women delegation members at the World Health Assembly per year by Government Effectiveness indicator 2019. (c) Estimated years ± standard error (SE) until gender parity (45%-55% inferred women) from 2010-2019 by Government Effectiveness indicator 2019. *Note, only countries with trend in estimated change of % women delegation members per year adjusted P-value <0.05 were included.* The Government Effectiveness Worldwide governance indicator is a reflection of the perceived public services quality, civil service quality and degree of independence from political pressure, policy formulation and implementation quality, and the credibility of government's commitment to policies. Estimates of governance performance on these indicators ranges from 2.5 (strong) to -2.5 (weak).[4,5]

van Daalen KR, *et al. BMJ Global Health* 2022; 7:e009312. doi: 10.1136/bmjgh-2022-009312

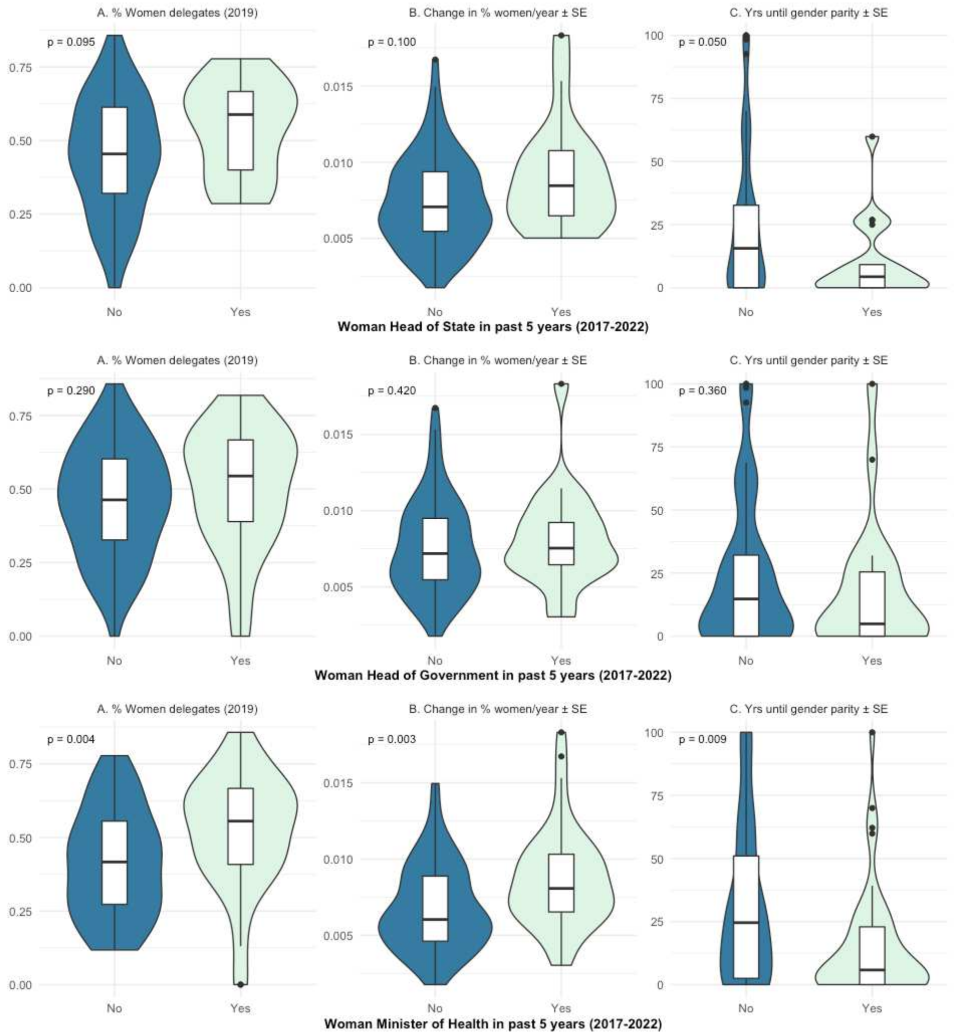

Supplement Figure 8. Distribution of gender representation in WHA delegations in countries with a woman HoS, HoG or MoH in the past five years (2017-2022). (a) Proportion (%) ± 95% Confidence Interval (CI) of inferred women delegation members at the World Health Assembly in 2019 by countries having a woman HoS, HoG or MoH in the past five years. (b) Estimated change (%) ± standard error (SE) of inferred women delegation members at the World Health Assembly per year by countries having a woman HoS, HoG or MoH in the past five years. (c) Estimated years ± standard error (SE) until gender parity (45%-55% inferred women) from 2010-2019 by countries having a woman HoS, HoG or MoH in the past five years. The non-parametric Wilcoxon signed-rank test was utilised to test for the difference in distributions – with a p-value <0.05 considered to be statistically significant.

Note, only countries with trend in estimated change of % women delegation members per year adjusted P-value <0.05 were included.

Supplement Table 1. Overview with documentation used to extract delegation members for each World Health Assembly (1-74).

#	Documentation reference
1	https://apps.who.int/iris/bitstream/handle/10665/85592/Official_record13_eng.pdf?sequence=1&isAllowed=y
2	https://apps.who.int/iris/handle/10665/85600?search-result=true&query=Second+world+health+assembly+verbatim+records&scope=&rpp=10&sort_by=score&order=desc
3	https://apps.who.int/iris/handle/10665/85607?search-result=true&query=Third+World+Health+Assembly+Verbatim+Records+Plenary&scope=&rpp=10&sort_by=score&order=desc
4	https://apps.who.int/iris/bitstream/handle/10665/85614/Official_record35_eng.pdf?sequence=1&isAllowed=y
5	https://apps.who.int/iris/bitstream/handle/10665/85641/Official_record42_eng.pdf?sequence=1&isAllowed=y
6	https://apps.who.int/iris/bitstream/handle/10665/85647/Official_record48_eng.pdf?sequence=1&isAllowed=y
7	https://apps.who.int/iris/handle/10665/85654?search-result=true&query=Seventh+World+Health+Assembly+verbatim+records&scope=%2F&filtertype_0=dateIssued&filter_relational_operator_0=equals&filter_0=%5B1920+TO+1959%5D&rpp=10&sort_by=score&order=desc
8	https://apps.who.int/iris/bitstream/handle/10665/85662/Official_record63_eng.pdf?sequence=1&isAllowed=y
9	https://apps.who.int/iris/handle/10665/85678?search-result=true&query=Second+World+Health+Assembly%2C+Geneva+24+June+to+24+July+1948%3A+plenary+meetings%3A+verbatim+records%3A+main+committees%3A+summary+of+resolutions+and+decisions&scope=%2F&rpp=10&sort_by=score&order=desc&page=1
10	https://apps.who.int/iris/handle/10665/85686?search-result=true&query=Second+World+Health+Assembly%2C+Geneva+24+June+to+24+July+1948%3A+plenary+meetings%3A+verbatim+records%3A+main+committees%3A+summary+of+resolutions+and+decisions&scope=%2F&rpp=10&sort_by=score&order=desc&page=1
11	https://apps.who.int/iris/handle/10665/85706?search-result=true&query=Second+World+Health+Assembly%2C+Geneva+24+June+to+24+July+1948%3A+plenary+meetings%3A+verbatim+records%3A+main+committees%3A+summary+of+resolutions+and+decisions&scope=%2F&rpp=10&sort_by=score&order=desc&page=2
12	https://apps.who.int/iris/handle/10665/85719?search-result=true&query=Second+World+Health+Assembly%2C+Geneva+24+June+to+24+July+1948%3A+plenary+meetings%3A+verbatim+records%3A+main+committees%3A+summary+of+resolutions+and+decisions&scope=%2F&rpp=10&sort_by=score&order=desc&page=1
13	https://apps.who.int/iris/handle/10665/85729?search-result=true&query=Second+World+Health+Assembly%2C+Geneva+24+June+to+24+July+1948%3A+plenary+meetings%3A+verbatim+records%3A+main+committees%3A+summary+of+resolutions+and+decisions&scope=%2F&rpp=10&sort_by=score&order=desc&page=2

14	https://apps.who.int/iris/handle/10665/85738?search-result=true&query=Second+World+Health+Assembly%2C+Geneva+24+June+to+24+July+1948%3A+plenary+meetings%3A+verbatim+records%3A+main+committees%3A+summary+of+resolutions+and+decisions&scope=%2F&rpp=10&sort_by=score&order=desc&page=2
15	https://apps.who.int/iris/handle/10665/85749?search-result=true&query=Second+World+Health+Assembly%2C+Geneva+24+June+to+24+July+1948%3A+plenary+meetings%3A+verbatim+records%3A+main+committees%3A+summary+of+resolutions+and+decisions&scope=%2F&rpp=10&sort_by=score&order=desc&page=2
16	https://apps.who.int/iris/bitstream/handle/10665/85759/Official_record128_eng.pdf?sequence=1&isAllowed=y
17	https://apps.who.int/iris/handle/10665/85770?search-result=true&query=Second+World+Health+Assembly%2C+Geneva+24+June+to+24+July+1948%3A+plenary+meetings%3A+verbatim+records%3A+main+committees%3A+summary+of+resolutions+and+decisions&scope=%2F&rpp=10&sort_by=score&order=desc&page=2
18	https://apps.who.int/iris/bitstream/handle/10665/85781/Official_record144_eng.pdf?sequence=1&isAllowed=y
19	https://apps.who.int/iris/handle/10665/85789?search-result=true&query=Nineteenth+World+Health+Assembly+verbatim+records&scope=&rpp=10&sort_by=score&order=desc
20	https://apps.who.int/iris/handle/10665/85801?search-result=true&query=Twentieth+world+health+assembly+verbatim+records&scope=%2F&rpp=10&sort_by=score&order=desc
21	https://apps.who.int/iris/handle/10665/85809?search-result=true&query=twenty+first+world+health+assembly+verbatim+records&scope=&rpp=10&sort_by=score&order=desc
22	https://apps.who.int/iris/bitstream/handle/10665/85817/Official_record177_eng.pdf?sequence=1&isAllowed=y
23	https://apps.who.int/iris/bitstream/handle/10665/85825/Official_record185_eng.pdf?sequence=1&isAllowed=y
24	https://apps.who.int/iris/handle/10665/85835?search-result=true&query=Twenty+fourth+World+Health+Assembly+verbatim+records&scope=%2F&rpp=10&sort_by=score&order=desc
25	https://apps.who.int/iris/handle/10665/85851?search-result=true&query=Twenty+fifth+World+Health+Assembly+verbatim+records&scope=%2F&rpp=10&sort_by=score&order=desc
26	https://apps.who.int/iris/bitstream/handle/10665/85863/Official_record210_eng.pdf?sequence=1&isAllowed=y
27	https://apps.who.int/iris/bitstream/handle/10665/85875/Official_record218_eng.pdf?sequence=1&isAllowed=y
28	https://apps.who.int/iris/handle/10665/86023?search-result=true&query=Twenty+eigth+World+Health+Assembly+verbatim+records&scope=%2F&rpp=10&sort_by=score&order=desc
29	https://apps.who.int/iris/handle/10665/95341?search-result=true&query=29+world+health+assembly+ACTAS+OFICIALES+DE+LA+ORGANIZACION+MUNDIAL+DE+LA+SALUD+N%C2%B0+234&scope=%2F&rpp=10&sort_by=score&order=desc
30	https://apps.who.int/iris/handle/10665/86037?search-result=true&query=Twenty+ninth+World+Health+Assembly+verbatim+records&scope=%2F&rpp=10&sort_by=score&order=desc&page=4
31	https://apps.who.int/iris/handle/10665/86044?search-result=true&query=Thirty+first+world+health+assembly+verbatim+records&scope=&rpp=10&sort_by=score&order=desc
32	https://apps.who.int/iris/bitstream/handle/10665/153658/WHA32_1979-REC-1_eng.pdf?sequence=1&isAllowed=y

33	https://apps.who.int/iris/bitstream/handle/10665/154893/WHA33_1980-REC-1_eng.pdf?sequence=1&isAllowed=y
34	https://apps.who.int/iris/bitstream/handle/10665/155679/WHA34_1981-REC-1_eng.pdf?sequence=1&isAllowed=y
35	https://apps.who.int/iris/bitstream/handle/10665/156782/WHA35_1982-REC-1_eng.pdf?sequence=1&isAllowed=y
36	https://apps.who.int/iris/bitstream/handle/10665/159886/WHA36_1983-REC-1_eng.pdf?sequence=1&isAllowed=y
37	https://apps.who.int/iris/bitstream/handle/10665/160772/WHA37_1984-REC-1_eng.pdf?sequence=1&isAllowed=y
38	https://apps.who.int/iris/bitstream/handle/10665/161291/WHA38_1985-REC-1_eng.pdf?sequence=1&isAllowed=y
39	https://apps.who.int/iris/handle/10665/162252?search-result=true&query=WHA39%2F+1986%2FREC%2F+1&scope=%2F&rpp=10&sort_by=score&order=desc
40	https://apps.who.int/iris/bitstream/handle/10665/163838/WHA40_1987-REC-1_eng.pdf?sequence=1&isAllowed=y
41	https://apps.who.int/iris/bitstream/handle/10665/164197/WHA41_1988-REC-1_eng.pdf?sequence=1&isAllowed=y
42	https://apps.who.int/iris/handle/10665/171212?search-result=true&query=Forty+second+World+Health+Assembly+verbatim+records&scope=%2F&rpp=10&sort_by=score&order=desc
43	https://apps.who.int/iris/handle/10665/173423?search-result=true&query=Forty+third+World+Health+Assembly+verbatim+records&scope=%2F&rpp=10&sort_by=score&order=desc
44	https://apps.who.int/iris/handle/10665/173860?search-result=true&query=WHA44%2F1991%2FREC%2F2&scope=&rpp=10&sort_by=score&order=desc
45	https://apps.who.int/iris/handle/10665/175630?search-result=true&query=WHA45%2F1992%2FREC%2F1&scope=&rpp=10&sort_by=score&order=desc
46	https://apps.who.int/iris/handle/10665/176262?search-result=true&query=wha46%2F1993%2Frec%2F1&scope=&rpp=10&sort_by=score&order=desc
47	https://apps.who.int/iris/handle/10665/177049?search-result=true&query=WHA47%2F1994%2FREC%2F1&scope=&rpp=10&sort_by=score&order=desc
48	https://apps.who.int/iris/handle/10665/178296?search-result=true&query=WHA48%2F1995%2FREC%2F1&scope=&rpp=10&sort_by=score&order=desc
49	https://apps.who.int/iris/bitstream/handle/10665/203895/WHA49_1996-REC-1_spa.pdf?sequence=1&isAllowed=y
50	https://apps.who.int/iris/bitstream/handle/10665/179638/WHA50_1997-REC-1_eng.pdf?sequence=1&isAllowed=y
51	https://apps.who.int/iris/bitstream/handle/10665/258905/WHA51-1998-REC-2-eng-fre.pdf?sequence=1&isAllowed=y
52	https://apps.who.int/iris/bitstream/handle/10665/258945/WHA52-1999-REC-2-eng-fre.pdf?sequence=1&isAllowed=y
53	https://apps.who.int/iris/bitstream/handle/10665/258949/WHA53-2000-REC-2-eng-fre.pdf?sequence=1&isAllowed=y
54	https://apps.who.int/iris/bitstream/handle/10665/258950/WHA54-2001-REC-2-eng-fre.pdf?sequence=1&isAllowed=y
55	https://apps.who.int/iris/bitstream/handle/10665/258959/WHA55-2002-REC-2-eng-fre.pdf?sequence=1&isAllowed=y

56	https://apps.who.int/iris/bitstream/handle/10665/258978/WHA56-2003-REC-2-eng-fre.pdf?sequence=1&isAllowed=y
57	https://apps.who.int/iris/bitstream/handle/10665/260146/WHA57-2004-REC-2-eng-fre.pdf?sequence=1&isAllowed=y
58	https://apps.who.int/iris/bitstream/handle/10665/260170/WHA58-2005-REC-2-eng-fre.pdf?sequence=1&isAllowed=y
59	https://apps.who.int/iris/bitstream/handle/10665/260171/WHA59-2006-REC-2-eng-fre.pdf?sequence=1&isAllowed=y
60	https://apps.who.int/iris/bitstream/handle/10665/22627/A60_DIV1R1.pdf?sequence=1&isAllowed=y
61	https://apps.who.int/iris/bitstream/handle/10665/23570/A61_REC2.pdf?sequence=1&isAllowed=y
62	https://apps.who.int/iris/handle/10665/2263?search-result=true&query=WHA62%2F2009%2FREC%2F2&scope=&rpp=10&sort_by=score&order=desc
63	https://apps.who.int/iris/handle/10665/3105?search-result=true&query=WHA63%2F2010%2FREC%2F2&scope=%2F&rpp=10&sort_by=score&order=desc
64	https://apps.who.int/iris/bitstream/handle/10665/4462/A64_DIV1_R1.pdf?sequence=1&isAllowed=y
65	https://apps.who.int/iris/handle/10665/80052?search-result=true&query=A65%2FDIV%2F1+Rev.1&scope=&rpp=10&sort_by=score&order=desc
66	https://apps.who.int/iris/handle/10665/150533?search-result=true&query=A66%2FDIV%2F1+Rev.1&scope=&rpp=10&sort_by=score&order=desc
67	https://apps.who.int/iris/handle/10665/162733?search-result=true&query=A67%2FDIV%2F1+Rev.1+sixty-seventh&scope=%2F&rpp=10&sort_by=score&order=desc
68	https://apps.who.int/iris/handle/10665/260360?search-result=true&query=SIXTY-EIGHTH+WORLD+HEALTH+ASSEMBLY+LIST+OF+DELEGATES+AND+OTHER+PARTICIPANTS+WHA68%2FDIV%2F1+Rev.1+25+May+2015&scope=&rpp=10&sort_by=score&order=desc
69	https://apps.who.int/iris/handle/10665/252771?search-result=true&query=a69%2Fdiv%2F1+rev.+1&scope=&rpp=10&sort_by=score&order=desc
70	https://apps.who.int/iris/handle/10665/275334?search-result=true&query=A70%2FDIV%2F1+Rev.1&scope=&rpp=10&sort_by=score&order=desc
71	https://apps.who.int/iris/handle/10665/277334?search-result=true&query=WHA71%2FDIV.%2F1+Rev.2&scope=&rpp=10&sort_by=score&order=desc&page=2
72	https://apps.who.int/iris/handle/10665/329202?search-result=true&query=A72%2FDIV.%2F1&scope=&rpp=10&sort_by=score&order=desc
73	https://apps.who.int/gb/ebwha/pdf_files/WHA73/A73_DIV1REV1-en.pdf
74	https://apps.who.int/gb/ebwha/pdf_files/WHA74/A74_Div1Rev1-en.pdf

Supplement Table 2. Overview of gendered prefixes/names used for inferring likely gender based on the list of delegates provided in WHO documentation.

Men's prefixes	Women's Prefixes	Not gendered
Baron	Dame	Abbot
Comte	Datin	Admiral
Dato	Dra. (Spanish)	Archbishop
King	First Lady	Assoc. Prof.
Lord	Lady	Brigadier
M. (French)	Miss	Captain
Monsignor	Mlle. (French)	Cardinal
Mr.	Mme. (French)	Colonel (Col.)
Pehin	Mrs.	Comte
Prince	Ms.	Count
Rev. Father	Princess	Dr.
Shk.	Profesora (Spanish)	Dr. Jur.
Sir.	Queen	H.E.
Sr.	Sister	Inspecteur General
Sultan	Sra. (Spanish)	Lt. Col.
Tan Sri	Srta. (Spanish)	Prof.
		Reverend
		Senator
		Surgeon General
		The Hon.
		Vice Amiral

Supplement Table 3. Recoding of (former) countries, territories and political parties including their geopolitical context.

**Recoded (former) country names**		
**Extracted**	**Recoded as**	**Geopolitical context**
Burma	Myanmar	"Union of Burma" was renamed "Union of Myanmar" in 1989 by the military government. Myanmar being the Burmese word for Burma.[6]
Ceylon	Sri Lanka	In 1948, the colony of Ceylon gained independence from the British. In 1972 Ceylon was renamed the "Republic of Sri Lanka."[6]
Congo-Brazzaville	Republic of the Congo	When the French colonized this region, Brazzaville (the region's capital) was used in the name to distinguish from the DRC.[7]
Congo-Leopoldville	Democratic Republic of the Congo (DRC)	The DRC was named in 1964, but the capital name Leopoldville was used to distinguish from the Republic of the Congo. Leopoldville was renamed Kinshasa in 1966.[8]
Dahomey	Benin	Dahomey was a kingdom in Benin, but the name formally changed to the "Republic of Benin" in 1990.[6]
Democratic Kampuchea	Cambodia	Between 1975 and 1979, Democratic Kampuchea was the name given to Marxist-Leninist controlled Cambodia.[9]
Federation of Nigeria	Nigeria	From 1954-1960, the Federation of Nigeria was the name of the British protectorate until the country gained formal independence in 1960.[6]
Former Yugoslav Rep of Macedonia	Republic of North Macedonia	In 1991, the Republic of Macedonia (which was renamed as the Republic of North Macedonia in 2019) became one of the successor states of Yugoslavia.[6]
Gold Coast	Ghana	The Gold Coast was a former British colony and was renamed Ghana upon independence in 1957.[6]
Khmer Republic	Cambodia	The Khmer Republic was declared after a military coup in 1970. It fell in 1975 and the Kingdom of Cambodia was restored briefly until1976 when it became Democratic Kampuchea.[9]
Libyan Arab Jamahiriya & Libyan Arab Republic	Libya	Libya's name changed several times while Muammar Gaddafi was in power. From 1969-1977, it was the Libyan Arab Republic. In 1977, it became the Socialist People's Libyan Arab Jamahiriya. In 1986, it was known as the Great Socialist People's Libyan Arab Jamahirya. The fall of the last pro-Gaddafi site and Gaddafi's assassination in 2011 marked the end of the Libyan Arab Jamahirya.[6 10]
Northern Rhodesia	Zambia	Northern Rhodesia was a British protectorate in South Central Africa, and became independent in 1964 as Zambia.[6]
Palestine Liberation Organization	Palestine	The Palestinian Liberation Organization, founded in 1964, serves as the official government of the State of Palestine and has received UN observer status since 1974.[11]
People's Republic of the Congo	Republic of the Congo	The People's republic of Congo was a Marxist-Leninist one-party socialist state between 1969-1992 in the Republic of the Congo.[7]
Siam	Thailand	Siam was the historical name for the Kingdom of Thailand until 1939. It was briefly known as Siam again from 1946-1948 and then reverted to Thailand in 1948.[12]
Southern Rhodesia	Zimbabwe	A land locked self-governing colony of the Britain that existed from 1932 to 1979 is now part of present-day Zimbabwe.[6]
Swaziland	Eswatini	Swaziland was the English name for the Kingdom of Eswatini. Swaziland was a British protectorate from 1903-1968. The name was officially changed to Eswatini (the Swazi language) in 2018.[6]

Tanganyika	Tanzania	The Kingdom of Tanganyika was renamed as Tanzania in 1964.[6]
Ukrainian Soviet Socialist Republic	Ukraine	Soviet Ukraine was one of the constituent republics of the Soviet Union from 1919-1991.[13]
Union of South Africa	South Africa	In 1910, the South Africa Act brought together the British Cape colonies as the Union of South Africa. It was dissolved and became the Republic of South Africa in 1961.[14]
Upper Volta	Burkina Faso	Upper Volta gained independence from the French in 1960 and renamed to Burkina Faso in 1984.[6]
Vatican City	Holy See	The Holy See describes the region under the jurisdiction of the Pope, which includes Vatican City.[15]
Western Samoa	Samoa	Samoa gained independence from New Zealand in 1962 and was known as Western Samoa until 1997.[6]
Zaire	Democratic Republic of the Congo	Following a coup in 1965, the country was renamed as the Republic of Zaire in 1971 until 1997 when its name reverted to the Democratic Republic of the Congo.[6]
Zanzibar	United Republic of Tanzania	In 1964, the United Republic of Tanganyika and Zanzibar was renamed as the United Republic of Tanzania, where Zanzibar remains as a self-governing state.[16]

Not recoded, including (former) countries, (former) territories, and (former) political party names

**Country, territory, political party**	**Geopolitical context**
African National Congress (South Africa)	South African political party was founded in 1912 as the South African Native National Congress. The main goal was to maintain voting rights for Black Africans and Coloured persons in Cape Province. In 1923 it was renamed as the African National Congress and from 1940 it focused on eliminating apartheid. Between 1960-1990 the ANC was banned. After the ban in 1994 Nelson Mandela (president of ANC) was elected the head of government.[17]
African National Congress (Zimbabwe)	The Southern Rhodesia African National Congress was a political party in Southern Rhodesia over 1957-1959. The party was committed to promoting the welfare of indigenous Africans.[18]
African Party for the Independence of Guinea and Cape Verde	Political party in Guinea-Bissau founded in 1954 (abbreviation PAIGC). It was originally formed to advocate for the independence from Portugal. Guinea-Bissau gained independence in 1973. PAIGC also governed Cape Verde from 1975 to 1980.[19]
Chinese Taipei	In most international forums and organisations, Taiwan (Republic of China) participates under the name “Chinese Taipei as compromise with the People’s Republic of China (PRC).[20]
Czechoslovakia	Czechoslovakia was a sovereign state in central Europe from 1918 until sometime between 1939-1945.[21]
Federation of Malaya	The Federation of Malaya unified the territories that were British colonies and existed between 1948 and 1963.[22]
Federation of Rhodesia and Nyasaland	The Federation of Rhodesia and Nyasaland, also known as the Central African Federation, was a colonial federation that consisted of three southern African territories: the self-governing British colony of Southern Rhodesia and the British protectorates of Northern Rhodesia and Nyasaland. It existed between 1953 and 1963.[6,23]
German Democratic Republic (East Germany)	East Germany, officially the German Democratic Republic, was a state that existed from 1949 to 1990 in eastern Germany as part of the Eastern Bloc in the Cold War.[24]
Germany - British Zone of Occupation	Post-World War II, the country was divided into four occupation zones from 1945 to 1949. In May 1949 the British, French, and American zones were joined to form the Federal Republic of Germany.[25]
Germany - French Zone of Occupation	Post-World War II, the country was divided into four occupation zones from 1945 to 1949. In May 1949 the British, French, and American zones were joined to form the Federal Republic of Germany.[25]
Germany - Americal Zone of Occupation	Post-World War II, the country was divided into four occupation zones from 1945 to 1949. In May 1949 the British, French, and American zones were joined to form the Federal Republic of Germany.[25]

Holy See	The Holy See is the universal government of the Catholic Church. It operates from Vatican City State which is an sovereign, independent territory. The Holy See has a permanent observer status at the United Nations.[15]
Morocco (French Zone)	The French protectorate of Morocco was the French military occupation of a large part of Morocco. The protectorate was officially established 30 March 1912, when Sultan Abd al-Hafid signed the Treaty of Fes. Independence was proclaimed in 1956.[26]
Morocco (Spanish Zone)	The Spanish protectorate in Morocco existed from 1912 to 1956 and is now part of present-day Morocco.[26]
Muscat and Oman	The Sultanate of Muscat and Oman, was a sovereign state that encompassed the present-day Oman and parts of present-day United Arab Emirates and Gwadar, Pakistan. It existed between 1856–1970.[27]
North Vietnam	North Vietnam was a socialist state in Southeast Asia from 1945 to 1976. Also called Democratic Republic of Vietnam (DRV).[28]
Order of Malta	Since 1113 the Sovereign Order of Malta is a lay religious order of the Catholic Church and a subject of international law. The Order of Malta has diplomatic relations with over 100 states and has a permanent observer status at the United Nations.[29]
Palestine	Palestine is considered to be a de jure sovereign state in Western Asia, comprising the Gaza strip, West Bank and parts of modern Israel.[22]
Pan Africanist Congress of Azania (South Africa)	The Pan Africanist Congress of Azania was a national liberation movement and now a political party, formally launched in 1959 that advocates for a South Africa based on African nationalism.[30]
Patriotic Front (Zimbabwe)	This was a coalition of two parties (the Zimbabwe African People’s Union (ZAPU) and the Zimbabwe African National Union (ZANU) which collaborated against White minority rule in Rhodesia.[31]
Puerto Rico	Puerto Rico is an unincorporated territory of the United States, but not an independent country or U.S. state.[32]
Ruanda-Urundi	Ruanda-Urundi (also Rwanda-Burundi) was a colonial territory ruled by Belgium from 1916-1962.[33]
Seychelles Democratic Party	Political party in the Seychelles that was founded in 1964. It governed Seychelles from 1976-1977.[34]
Seychelles Popular Party	Liberal political party in the Seychelles, which was formed in 1994 by the merger of the National Alliance Party, Parti Seselwa and the Seychelles National Movement.
Serbia and Montenegro	Serbia and Montenegro existed between 1992-2006, when it was dissolved after the breakup of Yugoslavia.[35]
South Vietnam	A part of present-day Vietnam that was a separate country from 1955 to 1975 during the Cold War.[28]
South Yemen	A country that existed from 1967-1990 in what are now the eastern provinces of present-day Republic of Yemen and the island of Socotra.[36]
Southern Korea (American Zone of Occupation)	The Southern half of the Korean Peninsula was governed by the United States Army Military Government in Korea from September 1945 to August 1948.[37]
Tokelau	Dependent territory of New Zealand, previously known as the Union Islands.[38]
Union of the Soviet Socialist Republics	The USSR was a communist state that existed from 1922 to 1991 and spanned Eurasia. Its dissolution led to multiple nation states including Armenia, Azerbaijan, Belarus, Estonia, Georgia, Kazakhstan, Kyrgyzstan, Latvia, Lithuania, Moldova, Russia, Tajikistan, Turkmenistan, Ukraine and Uzbekistan.[39]
United Arab Republic	Between 1958 and 1971, the United Arab Republic was a sovereign state in the Middle East - until 1961 this was a political relationship between Egypt and Syria, at which point Syria seceded after a coup. Egypt continued to be known as the United Arab Republic until 1971.[40]
Yugoslavia	A nation that was founded after WWI and was dissolved after a number of conflicts in the early 1990s. Was made up of what is present day Bosnia and Herzegovina, Croatia, Macedonia, Montenegro, Serbia (including the regions of Kosovo and Vojvodina) and Slovenia.[41]

Supplement Table 4. Classification of countries, territories and political parties in World Health Organization (WHO) region, United Nations (UN) region, World Bank region, World Bank income group and gender inequality index (GII).

Country	WHO region 2022[3]	UN region 2022[1]	World Bank region 2022[2]	World Bank income group 2022[2]	GII 2019[42]
Afghanistan	Eastern Mediterranean	Asia and Pacific	South Asia	Low-income	0.655
African National Congress (South Africa)	Africa*	Africa*	Sub-Saharan Africa*	NA	NA
African National Congress (Zimbabwe)	Africa*	Africa*	Sub-Saharan Africa*	NA	NA
African Party for the Independence of Guinea and Cape Verde	Africa*	Africa*	Sub-Saharan Africa*	NA	NA
Albania	Europe	Eastern European	Europe and Central Asia	Upper-middle-income	0.181
Algeria	Africa	Africa	Middle East and North Africa	Lower-middle-income	0.429
Andorra	Europe	Western European and Other	Europe and Central Asia	High-income	NA
Angola	Africa	Africa	Sub-Saharan Africa	Lower-middle-income	0.536
Antigua and Barbuda	Americas	Latin America and Caribbean	Latin America and Caribbean	High-income	NA
Argentina	Americas	Latin America and Caribbean	Latin America and Caribbean	Upper-middle-income	0.328
Armenia	Europe	Eastern European	Europe and Central Asia	Upper-middle-income	0.245
Australia	Western Pacific	Western European and Other	East Asia and Pacific	High-income	0.097
Austria	Europe	Western European and Other	Europe and Central Asia	High-income	0.069
Azerbaijan	Europe	Eastern European	Europe and Central Asia	Upper-middle-income	0.323
Bahamas	Americas	Latin America and Caribbean	Latin America and Caribbean	High-income	0.341
Bahrain	Eastern Mediterranean	Asia and Pacific	Middle East and North Africa	High-income	0.212
Bangladesh	South-East Asia	Asia and Pacific	South Asia	Lower-middle-income	0.537
Barbados	Americas	Latin America and Caribbean	Latin America and Caribbean	High-income	0.252
Belarus	Europe	Eastern European	Europe and Central Asia	Upper-middle-income	0.118
Belgium	Europe	Western European and Other	Europe and Central Asia	High-income	0.043
Belize	Americas	Latin America and Caribbean	Latin America and Caribbean	Lower-middle-income	0.415
Benin	Africa	Africa	Sub-Saharan Africa	Lower-middle-income	0.612

Bhutan	South-East Asia	Asia and Pacific	South Asia	Lower-middle-income	0.421
Bolivia	Americas	Latin America and Caribbean	Latin America and Caribbean	Lower-middle-income	0.417
Bosnia and Herzegovina	Europe	Eastern European	Europe and Central Asia	Upper-middle-income	0.149
Botswana	Africa	Africa	Sub-Saharan Africa	Upper-middle-income	0.465
Brazil	Americas	Latin America and Caribbean	Latin America and Caribbean	Upper-middle-income	0.408
Brunei Darussalam	Western Pacific	Asia and Pacific	East Asia and Pacific	High-income	0.255
Bulgaria	Europe	Eastern European	Europe and Central Asia	Upper-middle-income	0.206
Burkina Faso	Africa	Africa	Sub-Saharan Africa	Low-income	0.594
Burundi	Africa	Africa	Sub-Saharan Africa	Low-income	0.504
Cambodia	Western Pacific	Asia and Pacific	East Asia and Pacific	Lower-middle-income	0.474
Cameroon	Africa	Africa	Sub-Saharan Africa	Lower-middle-income	0.560
Canada	Americas	Western European and Other	North America	High-income	0.08
Cape Verde	Africa	Africa	Sub-Saharan Africa	Lower-middle-income	0.397
Central African Republic	Africa	Africa	Sub-Saharan Africa	Low-income	0.68
Chad	Africa	Africa	Sub-Saharan Africa	Low-income	0.71
Chile	Americas	Latin America and Caribbean	Latin America and Caribbean	High-income	0.247
China	Western Pacific	Asia and Pacific	East Asia and Pacific	Upper-middle-income	0.168
Chinese Taipei	Western Pacific*	Asia and Pacific*	East Asia and Pacific	High-income	NA
Colombia	Americas	Latin America and Caribbean	Latin America and Caribbean	Upper-middle-income	0.428
Comoros	Africa	Africa	Sub-Saharan Africa	Lower-middle-income	NA
Cook Islands	Western Pacific	Asia and Pacific	East Asia and Pacific	NA	NA
Costa Rica	Americas	Latin America and Caribbean	Latin America and Caribbean	Upper-middle-income	0.288
Cote d'Ivoire	Africa	Africa	Sub-Saharan Africa	Lower-middle-income	0.638
Croatia	Europe	Eastern European	Europe and Central Asia	High-income	0.116
Cuba	Americas	Latin America and Caribbean	Latin America and Caribbean	Upper-middle-income	0.304
Cyprus	Europe	Asia and Pacific	Europe and Central Asia	High-income	0.086

Czech Republic	Europe	Eastern European	Europe and Central Asia	High-income	0.136
Czechoslovakia	Europe*	Eastern European*	Europe and Central Asia*	NA	NA
Democratic People's Republic of Korea (North Korea)	South-East Asia	Asia and Pacific	East Asia and Pacific	Low-income	NA
Democratic Republic of Congo	Africa	Africa	Sub-Saharan Africa	Low-income	0.617
Denmark	Europe	Western European and Other	Europe and Central Asia	High-income	0.038
Djibouti	Eastern Mediterranean	Africa	Middle East and North Africa	Lower-middle-income	NA
Dominica	Americas	Latin America and Caribbean	Latin America and Caribbean	Upper-middle-income	NA
Dominican Republic	Americas	Latin America and Caribbean	Latin America and Caribbean	Upper-middle-income	0.455
Ecuador	Americas	Latin America and Caribbean	Latin America and Caribbean	Upper-middle-income	0.384
Egypt	Eastern Mediterranean	Africa	Middle East and North Africa	Lower-middle-income	0.449
El Salvador	Americas	Latin America and Caribbean	Latin America and Caribbean	Lower-middle-income	0.383
Equatorial Guinea	Africa	Africa	Sub-Saharan Africa	Upper-middle-income	NA
Eritrea	Africa	Africa	Sub-Saharan Africa	Low-income	NA
Estonia	Europe	Eastern European	Europe and Central Asia	High-income	0.086
Eswatini	Africa	Africa	Sub-Saharan Africa	Low-income	0.567
Ethiopia	Africa	Africa	Sub-Saharan Africa	Low-income	0.517
Faroe Islands	Europe*	Western European and Other*	Europe and Central Asia	High-income	NA
Federal Republic of Germany	Europe	Western European and Other	Europe and Central Asia	High-income	0.084
Federated States of Micronesia	Western Pacific	Asia and Pacific	East Asia and Pacific	Lower-middle-income	NA
Federation of Malaya	Western Pacific*	Asia and Pacific*	East Asia and Pacific*	NA	NA
Federation of Rhodesia and Nyasaland	Africa*	Africa*	Sub-Saharan Africa*	NA	NA
Fiji	Western Pacific	Asia and Pacific	East Asia and Pacific	Upper-middle-income	0.37
Finland	Europe	Western European and Other	Europe and Central Asia	High-income	0.047
France	Europe	Western European and Other	Europe and Central Asia	High-income	0.049
Gabon	Africa	Africa	Sub-Saharan Africa	Upper-middle-income	0.525
Gambia	Africa	Africa	Sub-Saharan Africa	Low-income	0.612

Georgia	Europe	Eastern European	Europe and Central Asia	Upper-middle-income	0.331
German Democratic Republic	Europe*	Eastern European*	Europe and Central Asia*	NA	NA
Germany - Americal Zone of Occupation	Europe*	Western European and Other*	Europe and Central Asia*	NA	NA
Germany - British Zone of Occupation	Europe*	Western European and Other*	Europe and Central Asia*	NA	NA
Germany - French Zone of Occupation	Europe*	Western European and Other*	Europe and Central Asia*	NA	NA
Ghana	Africa	Africa	Sub-Saharan Africa	Lower-middle-income	0.538
Greece	Europe	Western European and Other	Europe and Central Asia	High-income	0.116
Grenada	Americas	Latin America and Caribbean	Latin America and Caribbean	Upper-middle-income	NA
Guatemala	Americas	Latin America and Caribbean	Latin America and Caribbean	Upper-middle-income	0.479
Guinea	Africa	Africa	Sub-Saharan Africa	Low-income	NA
Guinea Bissau	Africa	Africa	Sub-Saharan Africa	Low-income	NA
Guyana	Americas	Latin America and Caribbean	Latin America and Caribbean	Upper-middle-income	0.462
Haiti	Americas	Latin America and Caribbean	Latin America and Caribbean	Lower-middle-income	0.636
Holy See	NA	NA	NA	NA	NA
Honduras	Americas	Latin America and Caribbean	Latin America and Caribbean	Lower-middle-income	0.423
Hungary	Europe	Eastern European	Europe and Central Asia	High-income	0.233
Iceland	Europe	Western European and Other	Europe and Central Asia	High-income	0.058
India	South-East Asia	Asia and Pacific	South Asia	Lower-middle-income	0.488
Indonesia	South-East Asia	Asia and Pacific	East Asia and Pacific	Lower-middle-income	0.48
Iraq	Eastern Mediterranean	Asia and Pacific	Middle East and North Africa	Upper-middle-income	0.577
Ireland	Europe	Western European and Other	Europe and Central Asia	High-income	0.093
Islamic Republic of Iran	Eastern Mediterranean	South Asia	Middle East and North Africa	Lower-middle-income	0.459
Israel	Europe	Western European and Other	Middle East and North Africa	High-income	0.109
Italy	Europe	Western European and Other	Europe and Central Asia	High-income	0.069
Jamaica	Americas	Latin America and Caribbean	Latin America and Caribbean	Upper-middle-income	0.396
Japan	Western Pacific	Asia and Pacific	East Asia and Pacific	High-income	0.094

BMJ Publishing Group Limited (BMJ) disclaims all liability and responsibility arising from any reliance placed on this supplemental material which has been supplied by the author(s)

Jordan	Eastern Mediterranean	Asia and Pacific	Middle East and North Africa	Upper-middle-income	0.45
Kazakhstan	Europe	Asia and Pacific	Europe and Central Asia	Upper-middle-income	0.19
Kenya	Africa	Africa	Sub-Saharan Africa	Lower-middle-income	0.518
Kiribati	Western Pacific	Asia and Pacific	East Asia and Pacific	Lower-middle-income	NA
Kuwait	Eastern Mediterranean	Asia and Pacific	Middle East and North Africa	High-income	0.242
Kyrgyzstan	Europe	Asia and Pacific	Europe and Central Asia	Lower-middle-income	0.369
Lao People's Democratic Republic	Western Pacific	Asia and Pacific	East Asia and Pacific	Lower-middle-income	0.459
Latvia	Europe	Eastern European	Europe and Central Asia	High-income	0.176
Lebanon	Eastern Mediterranean	Asia and Pacific	Middle East and North Africa	Upper-middle-income	0.411
Lesotho	Africa	Africa	Sub-Saharan Africa	Lower-middle-income	0.553
Liberation front of the Somalian Coast	Africa*	Africa*	Sub-Saharan Africa*	NA	NA
Liberia	Africa	Africa	Sub-Saharan Africa	Low-income	0.65
Libya	Eastern Mediterranean	Africa	Middle East and North Africa	Upper-middle-income	0.252
Lithuania	Europe	Eastern European	Europe and Central Asia	High-income	0.124
Luxembourg	Europe	Western European and Other	Europe and Central Asia	High-income	0.065
Madagascar	Africa	Africa	Sub-Saharan Africa	Low-income	NA
Malawi	Africa	Africa	Sub-Saharan Africa	Low-income	0.565
Malaysia	Western Pacific	Asia and Pacific	East Asia and Pacific	Upper-middle-income	0.253
Maldives	South-East Asia	Asia and Pacific	South Asia	Upper-middle-income	0.369
Mali	Africa	Africa	Sub-Saharan Africa	Low-income	0.671
Malta	Europe	Western European and Other	Middle East and North Africa	High-income	0.175
Marshall Islands	Western Pacific	Asia and Pacific	East Asia and Pacific	Upper-middle-income	NA
Mauritania	Africa	Africa	Sub-Saharan Africa	Lower-middle-income	0.634
Mauritius	Africa	Africa	Sub-Saharan Africa	Upper-middle-income	0.347
Mexico	Americas	Latin America and Caribbean	Latin America and Caribbean	Upper-middle-income	0.322
Moldova	Europe	Eastern European	Europe and Central Asia	Upper-middle-income	0.204

Monaco	Europe	Western European and Other	Europe and Central Asia	High-income	NA
Mongolia	Western Pacific	Asia and Pacific	East Asia and Pacific	Lower-middle-income	0.322
Montenegro	Europe	Eastern European	Europe and Central Asia	Upper-middle-income	0.109
Morocco	Eastern Mediterranean	Africa	Middle East and North Africa	Lower-middle-income	0.454
Morocco - French Zone	Eastern Mediterranean*	Africa*	Middle East and North Africa*	NA	NA
Morocco - Spanish Zone	Eastern Mediterranean*	Africa*	Middle East and North Africa*	NA	NA
Mozambique	Africa	Africa	Sub-Saharan Africa	Low-income	0.523
Muscat and Oman	Eastern Mediterranean*	Africa*	Middle East and North Africa*	NA	NA
Myanmar	South-East Asia	Asia and Pacific	East Asia and Pacific	Lower-middle-income	0.478
Namibia	Africa	Africa	Sub-Saharan Africa	Upper-middle-income	0.44
Nauru	Western Pacific	Asia and Pacific	East Asia and Pacific	High-income	NA
Nepal	South-East Asia	Asia and Pacific	South Asia	Lower-middle-income	0.452
Netherlands	Europe	Western European and Other	Europe and Central Asia	High-income	0.043
New Zealand	Western Pacific	Western European and Other	East Asia and Pacific	High-income	0.123
Nicaragua	Americas	Latin America and Caribbean	Latin America and Caribbean	Lower-middle-income	0.428
Niger	Africa	Africa	Sub-Saharan Africa	Low-income	0.642
Nigeria	Africa	Africa	Sub-Saharan Africa	Lower-middle-income	NA
Niue	Western Pacific	Asia and Pacific*	East Asia and Pacific*	NA	NA
North Vietnam	Western Pacific*	Asia and Pacific*	East Asia and Pacific*	NA	NA
Norway	Europe	Western European and Other	Europe and Central Asia	High-income	0.045
Oman	Eastern Mediterranean	Asia and Pacific	Middle East and North Africa	High-income	0.306
Order of Malta	NA	NA	NA	NA	NA
Pakistan	Eastern Mediterranean	Asia and Pacific	South Asia	Lower-middle-income	0.538
Palau	Western Pacific	Asia and Pacific	East Asia and Pacific	High-income	NA
Palestine	Europe*	Western European and Other*	Middle East and North Africa*	NA	NA
Pan Africanist Congress of Azania (South Africa)	Africa*	Africa*	Sub-Saharan Africa*	NA	NA

Panama	Americas	Latin America and Caribbean	Latin America and Caribbean	Upper-middle-income	0.407
Papua New Guinea	Western Pacific	Asia and Pacific	East Asia and Pacific	Lower-middle-income	0.725
Paraguay	Americas	Latin America and Caribbean	Latin America and Caribbean	Upper-middle-income	0.446
Patriotic Front (Zimbabwe)	Africa*	Africa*	Sub-Saharan Africa*	NA	NA
Peru	Americas	Latin America and Caribbean	Latin America and Caribbean	Upper-middle-income	0.395
Philippines	Western Pacific	Asia and Pacific	East Asia and Pacific	Lower-middle-income	0.43
Poland	Europe	Eastern European	Europe and Central Asia	High-income	0.115
Portugal	Europe	Western European and Other	Europe and Central Asia	High-income	0.075
Puerto Rico	Americas*	Western European and Other*	Latin America and Caribbean	High-income	0.204
Qatar	Eastern Mediterranean	Asia and Pacific	Middle East and North Africa	High-income	0.185
Republic of Congo	Africa	Africa	Sub-Saharan Africa	Lower-middle-income	0.57
Republic of Korea	South-East Asia	Asia and Pacific	East Asia and Pacific	High-income	0.064
Republic of North Macedonia	Europe	Eastern European	Europe and Central Asia	Upper-middle-income	0.143
Romania	Europe	Eastern European	Europe and Central Asia	Upper-middle-income	0.276
Ruanda-Urundi	Africa*	Africa*	Sub-Saharan Africa*	NA	NA
Russian Federation	Europe	Eastern European	Europe and Central Asia	Upper-middle-income	0.225
Rwanda	Africa	Africa	Sub-Saharan Africa	Low-income	0.402
Saint Kitts and Nevis	Americas	Latin America and Caribbean	Latin America and Caribbean	High-income	NA
Saint Lucia	Americas	Latin America and Caribbean	Latin America and Caribbean	Lower-middle-income	0.401
Saint Vincent and the Grenadines	Americas	Latin America and Caribbean	Latin America and Caribbean	Upper-middle-income	NA
Samoa	Western Pacific	Asia and Pacific	East Asia and Pacific	Lower-middle-income	0.36
San Marino	Europe	Western European and Other	Europe and Central Asia	High-income	NA
Sao Tome and Principe	Africa	Africa	Sub-Saharan Africa	Lower-middle-income	0.537
Saudi Arabia	Eastern Mediterranean	Asia and Pacific	Middle East and North Africa	High-income	0.252
Senegal	Africa	Africa	Sub-Saharan Africa	Lower-middle-income	0.533
Serbia	Europe	Eastern European	Europe and Central Asia	Upper-middle-income	0.132

Serbia and Montenegro	Europe*	Eastern European*	Europe and Central Asia*	NA	NA
Seychelles	Africa	Africa	Sub-Saharan Africa	High-income	NA
Seychelles Democratic Party	Africa*	Africa*	Sub-Saharan Africa*	NA	NA
Seychelles Popular Party	Africa*	Africa*	Sub-Saharan Africa*	NA	NA
Sierra Leone	Africa	Africa	Sub-Saharan Africa	Low-income	0.644
Singapore	South-East Asia	Asia and Pacific	East Asia and Pacific	High-income	0.065
Slovakia	Europe	Eastern European	Europe and Central Asia	High-income	0.191
Slovenia	Europe	Eastern European	Europe and Central Asia	High-income	0.063
Solomon Islands	Western Pacific	Asia and Pacific	East Asia and Pacific	Lower-middle-income	NA
Somalia	Western Pacific	Asia and Pacific	East Asia and Pacific	Lower-middle-income	NA
South Africa	Africa	Africa	Sub-Saharan Africa	Upper-middle-income	0.406
South Sudan	Africa	Africa	Sub-Saharan Africa	Low-income	NA
South Vietnam	Western Pacific*	Asia and Pacific*	East Asia and Pacific*	NA	NA
South Yemen	Eastern Mediterranean*	Asia and Pacific*	Middle East and North Africa*	NA	NA
Southern Korea - American Zone of Occupation	South-East Asia*	Asia and Pacific*	East Asia and Pacific*	NA	NA
Spain	Europe	Western European and Other	Europe and Central Asia	High-income	0.07
Sri Lanka	South-East Asia	Asia and Pacific	South Asia	Lower-middle-income	0.401
Sudan	Eastern Mediterranean	Africa	Sub-Saharan Africa	Low-income	0.545
Suriname	Americas	Latin America and Caribbean	Latin America and Caribbean	Upper-middle-income	0.436
Sweden	Europe	Western European and Other	Europe and Central Asia	High-income	0.039
Switzerland	Europe	Western European and Other	Europe and Central Asia	High-income	0.025
Syrian Arab Republic	Eastern Mediterranean	Asia and Pacific	Middle East and North Africa	Low-income	0.482
Tajikistan	Europe	Asia and Pacific	Europe and Central Asia	Lower-middle-income	0.314
Thailand	South-East Asia	Asia and Pacific	East Asia and Pacific	Upper-middle-income	0.359
Timor Leste	South-East Asia	Asia and Pacific	East Asia and Pacific	Lower-middle-income	NA
Togo	Africa	Africa	Sub-Saharan Africa	Low-income	0.573

Tokelau	Western Pacific*	Western European and Other*	East Asia and Pacific*	NA	NA
Tonga	Western Pacific	Asia and Pacific	East Asia and Pacific	Upper-middle-income	0.354
Trinidad and Tobago	Americas	Latin America and Caribbean	Latin America and Caribbean	High-income	0.323
Tunisia	Eastern Mediterranean	Africa	Middle East and North Africa	High-income	0.296
Turkey	Europe	Western European and Other	Europe and Central Asia	Upper-middle-income	0.306
Turkmenistan	Europe	Asia and Pacific	Europe and Central Asia	Upper-middle-income	NA
Tuvalu	Western Pacific	Asia and Pacific	East Asia and Pacific	Upper-middle-income	NA
Uganda	Africa	Africa	Sub-Saharan Africa	Low-income	0.535
Ukraine	Europe	Eastern European	Europe and Central Asia	Lower-middle-income	0.234
Union of the Soviet Socialist Republics	Europe*	Eastern European*	Europe and Central Asia*	NA	NA
United Arab Emirates	Eastern Mediterranean	Asia and Pacific	Middle East and North Africa	High-income	0.079
United Arab Republic	Eastern Mediterranean*	Asia and Pacific*	Middle East and North Africa*	NA	NA
United Kingdom	Europe	Western European and Other	Europe and Central Asia	High-income	0.118
United Republic of Tanzania	Africa	Africa	Sub-Saharan Africa	Lower-middle-income	0.556
United States of America	Americas	Western European and Other	North America	High-income	0.204
Uruguay	Americas	Latin America and Caribbean	Latin America and Caribbean	High-income	0.288
Uzbekistan	Europe	Asia and Pacific	Europe and Central Asia	Lower-middle-income	0.288
Vanuatu	Western Pacific	Asia and Pacific	East Asia and Pacific	Lower-middle-income	NA
Venezuela	Americas	Latin America and Caribbean	Latin America and Caribbean	NA⁺	0.479
Vietnam	Western Pacific	Asia and Pacific	East Asia and Pacific	Lower-middle-income	0.296
Yemen	Eastern Mediterranean	Asia and Pacific	Middle East and North Africa	Low-income	0.795
Yugoslavia	Europe*	Eastern European*	Europe and Central Asia*	NA	NA
Zambia	Africa	Africa	Sub-Saharan Africa	Lower-middle-income	0.539
Zimbabwe	Africa	Africa	Sub-Saharan Africa	Lower-middle-income	0.527

*Refer to former and current countries, territories or political parties for which we have extended the WHO region, UN region and World Bank region classifications based on their former or current geographical location.

⁺ Venezuela has been temporarily unclassified in the World Bank income groupings, pending revised national accounts statistics (per July 2021).[2]

Table 5. Included Worldwide Governance Indicators (World Bank).[4 5]

Country / Territory	Voice and Accountability Worldwide Governance Indicator (2019)	Government Effectiveness Worldwide Governance Indicator (2019)
Afghanistan	-1.006792426109314	-1.4630650281906128
Albania	0.14271074533462524	-6.2213201075792313E-2
Algeria	-1.056199312210083	-0.51639986038208008
Antigua and Barbuda	0.73324227333068848	-4.1233780793845654E-3
Argentina	0.57400608062744141	-8.6998365819454193E-2
Armenia	5.5476590991020203E-2	-6.6646300256252289E-2
Australia	1.2674413919448853	1.5715552568435669
Austria	1.3387953042984009	1.5277369022369385
Azerbaijan	-1.5295077562332153	-0.14017026126384735
Bahrain	-1.4292947053909302	0.29849055409431458
Bangladesh	-0.74182093143463135	-0.74028491973876953
Belgium	1.3102433681488037	1.1481317281723022
Bhutan	7.4757985770702362E-2	0.31243914365768433
Bolivia	-0.11432570219039917	-0.69932210445404053
Botswana	0.49996182322502136	0.42825746536254883
Brazil	0.29746577143669128	-0.186902716755867
Bulgaria	0.35563969612121582	0.26338973641395569
Burkina Faso	-0.20275132358074188	-0.75875735282897949
Burundi	-1.7234870195388794	-1.3264447450637817
Canada	1.4319146871566772	1.7312934398651123
Central African Republic	-1.2193750143051147	-1.7469326257705688
Chad	-1.4344154596328735	-1.5684139728546143
Chile	0.99114978313446045	1.0602893829345703
China	-1.6281181573867798	0.51700061559677124
Colombia	0.20484550297260284	7.0711903274059296E-2
Costa Rica	1.096335768699646	0.4225594699382782
Cuba	-1.4406877756118774	-0.17278853058815002
Cyprus	1.0486133098602295	0.99218153953552246
Denmark	1.5452375411987305	1.9102110862731934
El Salvador	0.11417551338672638	-0.46541833877563477
Federal Republic of Germany	1.3555911779403687	1.5311622619628906
Fiji	2.1415282040834427E-2	0.19961780309677124
Finland	1.564906120300293	2.006594181060791
France	1.1164376735687256	1.3721402883529663
Gabon	-1.0492092370986938	-0.89782404899597168
Georgia	0.16872023046016693	0.82873988151550293

Ghana	0.55790746212005615	-0.21064327657222748
Greece	0.81240028142929077	0.34902435541152954
Guatemala	-0.33547094464302063	-0.67697829008102417
Honduras	-0.57272619009017944	-0.6119987964630127
Hungary	0.34397643804550171	0.49605315923690796
Iceland	1.3039740324020386	1.5179398059844971
Indonesia	0.13210906088352203	0.18205122649669647
Ireland	1.3050894737243652	1.291791558265686
Islamic Republic of Iran	-1.3869702816009521	-0.55372565984725952
Israel	0.66013967990875244	1.3253247737884521
Italy	0.90664374828338623	0.48317438364028931
Jamaica	0.61428910493850708	0.49398410320281982
Japan	0.93134206533432007	1.5863221883773804
Kenya	-0.31264817714691162	-0.38339018821716309
Kuwait	-0.65525496006011963	1.8212368711829185E-2
Latvia	0.85714906454086304	1.1047132015228271
Libya	-1.4748243093490601	-1.9222908020019531
Lithuania	0.9963756799697876	1.0430692434310913
Luxembourg	1.4935524463653564	1.7332957983016968
Madagascar	-0.24306319653987885	-1.1426148414611816
Malaysia	-4.9003709107637405E-2	0.99746757745742798
Mauritius	0.78070950508117676	0.8703991174697876
Mexico	7.9714525490999222E-3	-0.15678699314594269
Monaco	0.65803587436676025	NA
Mongolia	0.32375407218933105	-0.19543415307998657
Morocco	-0.64730942249298096	-0.11948017030954361
Netherlands	1.4854559898376465	1.8032723665237427
New Zealand	1.5352920293807983	1.6705572605133057
Nicaragua	-1.0954198837280273	-0.77085143327713013
Nigeria	-0.43453225493431091	-1.0888626575469971
Oman	-1.1543257236480713	0.25904110074043274
Pakistan	-0.86280936002731323	-0.67713558673858643
Panama	0.59518170356750488	6.4511820673942566E-2
Paraguay	5.8215837925672531E-2	-0.53434425592422485
Peru	0.2613716721534729	-6.7870549857616425E-2
Philippines	6.5084416419267654E-3	5.2008401602506638E-2
Poland	0.6731717586517334	0.53480100631713867
Portugal	1.1964657306671143	1.1686815023422241
Republic of Congo	-1.2791706323623657	-1.387316107749939

van Daalen KR, *et al. BMJ Global Health* 2022; 7:e009312. doi: 10.1136/bmjgh-2022-009312

Republic of Korea	0.75148493051528931	1.3760876655578613
Romania	0.51632511615753174	-0.16217932105064392
Saudi Arabia	-1.6597045660018921	0.30617403984069824
Senegal	0.23559823632240295	-5.8101300150156021E-2
South Africa	0.64362168312072754	0.36765611171722412
Spain	1.0377616882324219	1.0011752843856812
Sudan	-1.6515998840332031	-1.6220585107803345
Suriname	0.3643706738948822	-0.59028255939483643
Sweden	1.5609753131866455	1.7092572450637817
Switzerland	1.4970487356185913	1.9517581462860107
Thailand	-0.84680747985839844	0.35656705498695374
Togo	-0.74097734689712524	-0.91736841201782227
Tunisia	0.25651702284812927	-0.10294915735721588
Turkey	-0.83429986238479614	4.788932204246521E-2
Uganda	-0.64435797929763794	-0.58546578884124756
United Arab Emirates	-1.1401491165161133	1.3768906593322754
United Kingdom	1.2613987922668457	1.48273766040802
United Republic of Tanzania	-0.52488887310028076	-0.87934422492980957
Venezuela	-1.4283932447433472	-1.6584689617156982
Vietnam	-1.4056812524795532	3.8309685885906219E-2

The Worldwide Governance Indicators are a set of aggregate indicators that combine the views of a large numbers of survey respondents (enterprises, citizens) – combining 30 individual data sources produced by various non-governmental organisations, international organisations, survey institutes, think thanks and private sector firms.

The Voice and Accountability Worldwide Governance Indicator is "*a reflection of the perceived extent to which a country's citizens are able to participate in selecting their government, freedom of expression, freedom of association and free media*." [4,5]

The Government Effectiveness Worldwide Governance Indicator is "*a reflection of the perceived public services quality, civil service quality and degree of independence from political pressure, policy formulation and implementation quality, and the credibility of government's commitment to policies*". [4,5]

Information on the methodology can be found in Kaufmann *et al.* 2010[5] and http://info.worldbank.org/governance/wgi/

Supplement Table 6. Summary of the data characteristics.

Data	
Membership to the WHA **(total N = 75,815)**	**% (N)**
Member State	98.17% (74,430)
Associate Member	0.22% (167)
Observer	1,61% (1,218)
WHA delegation roles **(total N = 75,815)**	**% (N)**
Chief Delegate	13.18% (9,994)
Deputy Chief Delegate	4.36% (3,303)
Delegate	20.68% (15,678)
Alternate	46.74% (35,439)
Adviser	13.33% (10,105)
Observer	1.62% (1,231)
Other	0.09% (65)
Inferred gender delegate **(total N = 75,815)**	**% (N)**
Men	68.89% (52,230)
Women	25.34% (19,212)
Unknown	5.77% (4,373)
Inferred gender diversity (1948-2021) **(total N = 10,944)**	**% (N)**
Majority women (>55% women)	8.88% (972)
Gender parity (45-55% women)	8.26% (904)
Majority men (>55% men)	82.86% (9,068)
WHO Region of delegates **(total N = 78,815)**	**% (N)**
Africa	21.09% (15,993)
Americas	15.55% (11,792)
Eastern Mediterranean	11.37% (8,618)
Europe	31.87% (24,164)
South-East Asia	9.14% (6,933)
Western Pacific	10.20% (7,730)
Not applicable	0.77% (585)
Socio-economic status of delegate country **(total N = 75,815)**	**% (N)**
High-income	39.16% (29,689)
Upper-middle income	9.99% (7,573)
Lower-middle income	24.75% (18,762)
Low income	22.32% (16,925)
Not applicable	3.78% (2,866)
Other information	
Total number of unique countries, territories, political parties	228
Total number of delegations (1948-2021)	10944
Range of Gender Inequality Index 2019	0.025-0.795
Time period	1948-2021

Supplement Table 7. Woman head of state (HoS), head of government (HoG) or minister of health (MoH) over the past 5 years by country (2017-2022). Former countries, former territories and political parties are considered *not applicable*. Current territories with their own HoS, HoG and MoH are included. Note, the function of HoS has in many parliamentary countries a ceremonial function with a separate HoG (e.g. in the Britisch Monarch), whilst in presidential systems the HoS is often also the HoG. Some semi-presidential system have both a HoS and HoG functioning as *de facto* leaders.

Country	Head of State	Head of Government	Minister of Health
**Afghanistan**	No	No	No
**African National Congress (South Africa)**	*Not applicable*	*Not applicable*	*Not applicable*
**African National Congress (Zimbabwe)**	*Not applicable*	*Not applicable*	*Not applicable*
**African Party for the Independence of Guinea and Cape Verde**	*Not applicable*	*Not applicable*	*Not applicable*
**Albania**	No	No	Ogerta Manastirliu (2017)
**Algeria**	No	No	No
**Andorra**	No	No	No
**Angola**	No	No	Silvia Paula Valentim Lutucuta (2017-present)
**Antigua and Barbuda**	Queen Elizabeth II	No	No
**Argentina**	No	No	Carla Vizzotti (2021-present) Carolina Stanley (2018-2019)
**Armenia**	No	No	Anahit Avanesyan (2021-present)
**Australia**	Queen Elizabeth II	No	No
**Austria**	No	Brigitte Bierlein (2019-2020)	Brigitte Zarfl (2019-2020), Beate Hartinger-Klein (2017-2018)
**Azerbaijan**	No	No	No
**Bahamas**	Queen Elizabeth II (1952-present), Gov General Marguerite Pindling (2014-2019)	No	No
**Bahrain**	No	No	Faeqa bint Saeed Al Saleh (2015-present)
**Bangladesh**	No	Sheikh Hasina (2009-present)	No
**Barbados**	Sandra Mason (2021-present)	Mia Mottley (2018-2021)	No
**Belarus**	No	No	No
**Belgium**	No	Sophie Wilmès (2019-2020)	Maggie De Block (2014-2020)
**Belize**	Queen Elizabeth II (1952-present), Gov General Froyla Tzalam (2021-present)	No	No
**Benin**	No	No	No
**Bhutan**	No	No	Dasho Dechen Wangmo (2018-present)
**Bolivia**	Jeanine Áñez (2019-2020)	Jeanine Áñez (2019-2020)	Ariana Campero (2015-2018)
**Bosnia and Herzegovina**	No	No	No

**Botswana**	No	No	No
**Brazil**	No	No	No
**Brunei Darussalam**	No	No	No
**Bulgaria**	No	No	Asena Serbezova (2021-present)
**Burkina Faso**	No	No	Leonie Claudine Lougué Sorgho (2019-2021)
**Burundi**	No	No	Sabine Ntakarutimana (2010-2020)
**Cambodia**	No	No	No
**Cameroon**	No	No	No
**Canada**	Queen Elizabeth II (1952-present), Gov General Mary Simon (2017-2021) Gov General Julie Payette (2021-present)	No	Patty Hajdu (2019-2021), Ginette Petitpas Taylor (2017-2019), Jane Philpott (2015-2017)
**Cape Verde**	No	No	No
**Central African Republic**	No	No	No
**Chad**	No	No	No
**Chile**	Michelle Bachelet (2014-2018)	Michelle Bachelet (2014-2018)	María Begoña Yarza (2022-present), Carmen Castillo Taucher (2015-2018)
**China**	No	No	No
**Chinese Taipei (Taiwan)**	Tsai Ing-wen (2016-present)	No	No
**Colombia**	No	No	No
**Comoros**	No	No	Loub Yakout Zaïdou (2019-present), Fatima Rashid (2017-2019)
**Cook Islands**	Queen Elizabeth II (1952-present)	No	Rose Toki-Brown (2018-2021)
**Costa Rica**	No	No	Giselle Amador Munoz (2018-2019)
**Cote d'Ivoire**	No	No	No
**Croatia**	Kolinda Grabar-Kitarović (2015-2020)	No	No
**Cuba**	No	No	No
**Cyprus**	No	No	No
**Czech Republic**	No	No	No
**Czechoslovakia**	*Not applicable*	*Not applicable*	*Not applicable*
**Democratic People's Republic of Korea**	No	No	O Chun-bok (2019-2021)
**Democratic Republic of Congo**	No	No	No
**Denmark**	Queen Margrethe II (1972-present)	Mette Frederiksen (2019-present)	Ellen Trane Nørby (2016-2019)
**Djibouti**	No	No	No
**Dominica**	No	No	No
**Dominican Republic**	No	No	No
**Ecuador**	No	No	Ximena Garzón-Villalba (2021-present), Catalina Andramuño (2019-2020), María Verónica Espinosa Serrano (2017-2019)
**Egypt**	No	No	Hala Zayed (2018-present)

**El Salvador**	No	No	Violeta Menjívar (2014-2019),<br>Ana Orellana Bendek (2019-present)
**Equatorial Guinea**	No	No	No
**Eritrea**	No	No	Amna Nurhusein (2009-present)
**Estonia**	Kersti Kaljulaid (2016-2021)	Kaja Kallas (2021-present)	Riina Sikkut (2018-2019)
**Eswatini**	No	No	Lizzy Nkosi (2020-present)
**Ethiopia**	Sahle-Work Zewde (2018-present)	No	Lia Tadesse (2020-present)
**Faroe Islands**	Queen Margrethe II (1972-present)	No	Sirið Stenberg (2015-present)
**Federal Republic of Germany**	No	Angela Merkel	No
**Federated States of Micronesia**	No	No	No
**Federation of Malaya**	*Not applicable*	*Not applicable*	*Not applicable*
**Federation of Rhodesia and Nyasaland**	*Not applicable*	*Not applicable*	*Not applicable*
**Fiji**	No	No	Rosy Sofia Akbar (2018-present)
**Finland**	No	Sanna Marin (2019-present)	Hanna Sarkkinen (2021-present),<br>Aino-Kaisa Pekonen (2019-2021),<br>Pirkko Mattila (2016-2019)
**France**	No	No	Agnès Buzyn (2017-2020),<br>Marisol Touraine (2012-2017)
**Gabon**	No	Rose Christiane Raponda (2020-present)	No
**Gambia**	No	No	No
**Georgia**	Salome Zourabichvili (2018-present)	No	Ekaterine Tikaradze (2019-2021)
**German Democratic Republic (East Germany)**	*Not applicable*	*Not applicable*	*Not applicable*
**Germany - Americal Zone of Occupation**	*Not applicable*	*Not applicable*	*Not applicable*
**Germany - British Zone of Occupation**	*Not applicable*	*Not applicable*	*Not applicable*
**Germany - French Zone of Occupation**	*Not applicable*	*Not applicable*	*Not applicable*
**Ghana**	No	No	No
**Greece**	Katerina Sakellaropoulou (2020 - Present)	No	No
**Grenada**	Queen Elizabeth II (1952-present),<br>Gov General Cécile La Grenade, (2013 - Present)	No	No
**Guatemala**	No	No	Amelia Flores (2020-2021),<br>Lucrecia Hernández Mack (2016 - 2017)
**Guinea**	No	No	No
**Guinea Bissau**	No	No	Magda Robalo Correia E Silva<br>(2019 - 2020)

**Guyana**	No	No	Volda lawrence (2018 - 2020)
**Haiti**	No	No	Marie Gréta Roy Clement (2017 - Present)
**Holy See**	*Not applicable*	*Not applicable*	*Not applicable*
**Honduras**	Xiomara Castro (2022-Present)	Xiomara Castro (2022-Present)	No
**Hungary**	No	No	No
**Iceland**	No	Katrín Jakobsdóttir (2017 - Present)	No
**India**	No	No	No
**Indonesia**	No	No	No
**Iraq**	No	No	No
**Ireland**	No	No	No
**Islamic Republic of Iran**	No	No	No
**Israel**	No	No	No
**Italy**	No	No	No
**Jamaica**	Queen Elizabeth II (1952-present)	No	No
**Japan**	No	No	No
**Jordan**	No	No	No
**Kazakhstan**	No	No	Azhar Giniyat (2022-present)
**Kenya**	No	No	No
**Kiribati**	No	No	No
**Kuwait**	No	No	No
**Kyrgyzstan**	No	No	No
**Lao People's Democratic Republic**	No	No	No
**Latvia**	No	No	Ilze Viņķele (2019 - 2021), Anda Chaksha (2016 - 2019)
**Lebanon**	No	No	No
**Lesotho**	No	No	No
**Liberation front of the Somalian Coast**	*Not applicable*	*Not applicable*	*Not applicable*
**Liberia**	Ellen Johnson Sirleaf (2006-2018)	Ellen Johnson Sirleaf (2006-2018)	Wilhelmina Jallah (2015-2018), Bernice Dahn (2018-present)
**Libya**	No	No	No
**Lithuania**	Dalia Grybauskaitė (2009-2019)	Ingrida Šimonytė (2020-present)	No
**Luxembourg**	No	No	Paulette Lenert (2020-present),<br>Lydia Mutsch (2013-2018)
**Madagascar**	No	No	No
**Malawi**	No	No	Khumbize Kandodo Chiponda<br>(2020-present)
**Malaysia**	No	No	No
**Maldives**	No	No	No
**Mali**	No	No	Fanta Siby
**Malta**	No	No	No
**Marshall Islands**	No	No	No
**Mauritania**	No	No	No
**Mauritius**	No	No	No

**Mexico**	No	No	No
**Moldova**	Maia Sandu (2020-present)	Natalia Gavrilița (2021-present), Maia Sandu (2019)	Ala Nemerenco (2019; 2021-present), Viorica Dumbraveanu (2019-2020)
**Monaco**	No	No	No
**Mongolia**	No	No	No
**Montenegro**	No	No	Jelena Borovinić-Bojović (2020-present)
**Morocco**	No	No	No
**Morocco (French Zone)**	*Not applicable*	*Not applicable*	*Not applicable*
**Morocco (Spanish Zone)**	*Not applicable*	*Not applicable*	*Not applicable*
**Mozambique**	No	No	No
**Muscat and Oman**	NA	No	No
**Myanmar**	No	No	No
**Namibia**	No	Saara Kuugongelwa-Amadhila (2015-present)	No
**Nauru**	No	No	Isabella Dageago (2019-present)
**Nepal**	Bidya Devi Bhandari (2015-present)	No	No
**Netherlands**	No	No	Edith Schippers (2010-2017)
**New Zealand**	Queen Elizabeth II (1952-present), Gov General Cindy Kiro (2021-present), Gov General Patsy Reddy (2016-2021)	Jacinda Ardern (2017-present)	No
**Nicaragua**	No	No	Martha Veronica Reyes Alvarez (2020-present), Sonia Castro Gonzalez (2011-2020)
**Niger**	No	No	No
**Nigeria**	No	No	No
**Niue**	Queen Elizabeth II (1952-present), Gov General Cindy Kiro (2021-present), Gov General Patsy Reddy (2016-2021)	No	No
**North Vietnam**	*Not applicable*	*Not applicable*	*Not applicable*
**Norway**	No	No	Ingvild Kjerkol (2021-present)
**Oman**	No	No	No
**Order of Malta**	*Not applicable*	*Not applicable*	*Not applicable*
**Pakistan**	No	No	No
**Palau**	No	No	No
**Palestine**	No	No	Mai al-Kaila (2019-present)
**Pan Africanist Congress of Azania (South Africa)**	*Not applicable*	*Not applicable*	*Not applicable*
**Panama**	No	No	Rosario Turner (2019-2020)
**Papua New Guinea**	Queen Elizabeth II (1952-present)	No	No
**Paraguay**	No	No	No
**Patriotic Front (Zimbabwe)**	*Not applicable*	*Not applicable*	*Not applicable*
**Peru**	No	No	Pilar Elena Mazzetti Soler

			(2020-2021), Elizabeth Hinostroza (2019-2020), Zulema Thomas (2019), Silvia Pesah (2018-2019)
**Philippines**	No	No	No
**Poland**	No	No	No
**Portugal**	No	No	Marta Temido (2018-present)
**Puerto Rico**	*Not applicable*	*Not applicable*	*Not applicable*
**Qatar**	No	No	Hanan Mohamed Al Kuwari (2016-present)
**Republic of Congo**	No	No	No
**Republic of Korea**	No	No	No
**Republic of North Macedonia**	No	No	No
**Romania**	No	No	No
**Ruanda-Urundi**	*Not applicable*	*Not applicable*	*Not applicable*
**Russian Federation**	No	No	No
**Rwanda**	No	No	No
**Saint Kitts and Nevis**	Queen Elizabeth II (1952-present)	No	Akilah Byron-Nisbet (2020-Present)
**Saint Lucia**	Queen Elizabeth II (1952-present), Gov General Pearlette Louisy (1997-2017)	No	Mary Isaac (2016-present)
**Saint Vincent and the Grenadines**	Queen Elizabeth II (1952-present), Gov General Susan Dougan (2019-present)	No	No
**Samoa**	No	Fiamē Naomi Mataʻafa (2021-present)	Faimalotoa Kika Stowers (2019-2021)
**San Marino**	Grazia Zafferani (2020), Mariella Mularoni (2019), Mimma Zavoli (2017), Vanessa D'Ambrosio (2017) *two captains regents elected every 6 months	N/A	No
**Sao Tome and Principe**	No	No	No
**Saudi Arabia**	No	No	No
**Senegal**	No	No	Awa Marie Coll-Seck (2012-2017)
**Serbia**	No	Ana Brnabić (2017-present)	No
**Serbia and Montenegro**	*Not applicable*	*Not applicable*	*Not applicable*
**Seychelles**	No	No	Peggy Vidot (2020-present)
**Seychelles Democratic Party**	*Not applicable*	*Not applicable*	*Not applicable*
**Seychelles Popular Party**	*Not applicable*	*Not applicable*	*Not applicable*
**Sierra Leone**	No	No	No
**Singapore**	Halimah Yacob (2017 - Present)	No	No
**Slovakia**	Zuzana Čaputová (2019 - Present)	No	No
**Slovenia**	No	No	No
**Solomon Islands**	Queen Elizabeth II (1952-present)	No	No
**Somalia**	No	No	Fawziya Abikar Nur (2017 - Present)

van Daalen KR, *et al. BMJ Global Health* 2022; 7:e009312. doi: 10.1136/bmjgh-2022-009312

**South Africa**	No	No	No
**South Sudan**	No	No	Elizabeth Achuei (2021 - 2022), Awel Deng (2022 - Present)
**South Vietnam**	*Not applicable*	*Not applicable*	*Not applicable*
**South Yemen**	*Not applicable*	*Not applicable*	*Not applicable*
**Southern Korea – American Zone of Occupation**	*Not applicable*	*Not applicable*	*Not applicable*
**Spain**	No	No	Carolina Darias (2021 - Present), María Luisa Carcedo (2018 - 2020), Carmen Montón (2018), Dolors Montserrat (2016 - 2018)
**Sri Lanka**	No	No	Pavithra Wanniarachchi (2019-2021)
**Sudan**	Raja Nicola (2019 - 2021), Aisha Musa el-Said (2019 - 2021), (Member of Collective HoS)	No	Sara Abdelazeem (2020 - 2021)
**Suriname**	No	No	No
**Sweden**	No	Magdalena Andersson (2021 - Present)	Lena Hallengren (2019-Present)
**Switzerland**	Simonetta Sommaruga (2020), Viola Amherd (2019 - Present), Karin Keller-Sutter (2019 - Present), Doris Leuthard (2006 - 2018), (Swiss Federal Council)	Doris Leuthard (2016 - 2017)	No
**Syrian Arab Republic**	No	No	No
**Tajikistan**	No	No	No
**Tanganyika**	*Not applicable*	*Not applicable*	*Not applicable*
**Thailand**	No	No	No
**Timor Leste**	No	No	Odete Maria Freitas Belo (2020 - Present)
**Togo**	No	Victoire Tomegah Dogbé (2020 - Present)	No
**Tokelau**	Queen Elizabeth II	No	No
**Tonga**	No	No	No
**Trinidad and Tobago**	Paula-Mae Weekes	No	No
**Tunisia**	No	Najla Bouden	No
**Turkey**	No	No	No
**Turkmenistan**	No	No	No
**Tuvalu**	Queen Elizabeth II (1952-present), Gov General Teniku Talesi Honolulu (2019-2021)	No	No
**Uganda**	No	Robinah Nabbanja (2021 - Present)	Jane Aceng
**Ukraine**	No	No	Zoriana Skaletska (2019-2020), Ulana Suprun (2016-2019)
**Union of the Soviet Socialist Republics**	*Not applicable*	*Not applicable*	*Not applicable*
**United Arab Emirates**	No	No	No
**United Arab Republic**	*Not applicable*	*Not applicable*	*Not applicable*

**United Kingdom**	Queen Elizabeth II	Theresa May (2016-2019)	No
**United Republic of Tanzania**	Samia Suluhu Hassan (2021-present)	Samia Suluhu Hassan (2021-present)	Ummy Mwalimu (2022-present; 2015-2020), Dorothy Gwajima (2020-2022)
**United States of America**	No	No	No
**Uruguay**	No	No	No
**Uzbekistan**	No	No	No
**Vanuatu**	No	No	No
**Venezuela**	No	No	Antonieta Caporale Zamora (2017)
**Vietnam**	Đặng Thị Ngọc Thịnh (2016 - 2021)	No	Nguyễn Thị Kim Tiến (2019 - 2021)
**Yemen**	No	No	No
**Yugoslavia**	*Not applicable*	*Not applicable*	*Not applicable*
**Zambia**	No	No	No
**Zimbabwe**	No	No	No

van Daalen KR, *et al. BMJ Global Health* 2022; 7:e009312. doi: 10.1136/bmjgh-2022-009312

References

1. Regional groups of Member States: United Nations; [Available from: https://www.un.org/dgacm/en/content/regional-groups accessed 6 March 2022.

2. The World by Income and Region: World Bank; [Available from: https://datatopics.worldbank.org/world-development-indicators/the-world-by-income-and-region.html accessed 6 March 2022.

3. Countries: World Health Organization; [Available from: https://www.who.int/countries accessed 6 March 2022.

4. Bank W. Worldwide Governance Indicators [Available from: http://info.worldbank.org/governance/wgi/ accessed 3 April 2022.

5. Kaufmann D, Kraay A, Mastruzzi M. The worldwide governance indicators: Methodology and analytical issues1. *Hague journal on the rule of law* 2011;3(2):220-46.

6. Timeline of country name changes in HMG use: 1919 to 2020: UK Ministry of Defence; [updated 14 May 2021. Available from: https://www.gov.uk/government/publications/country-names/country-name-changes-in-hmg-use-1919-to-20202022.

7. Cordell DD. Republic of the Congo. *Encyclopedia Britannica* 2021 2 June 2021. https://www.britannica.com/place/Republic-of-the-Congo.

8. Payanzo N, Lemarchand R, Cordell DD, et al. Democratic Republic of the Congo. *Encyclopedia Britannica* 2021 29 April 2021. https://www.britannica.com/place/Democratic-Republic-of-the-Congo.

9. Chandler DP, Overton LC. Cambodia. *Encyclopedia Britannica* 2021 10 March 2021. https://www.britannica.com/place/Cambodia.

10. Fowler G, Barbour N, Brown LC, et al. Libya. *Encyclopedia Britannica* 2022 31 March 2022. https://www.britannica.com/place/Libya.

11. Britannica TEoE. Palestine Liberation Organization. *Encyclopedia Britannica* 2020 15 July 2020. https://www.britannica.com/topic/Palestine-Liberation-Organization.

12. History of Thailand [Available from: https://www.nationsonline.org/oneworld/History/Thailand-history.htm.

13. Markus V, Stebelsky I. Ukranian Soviet Socialist Republic 1993 [Available from: http://www.encyclopediaofukraine.com/display.asp?linkpath=pages%5CU%5CK%5CUkrainianSovietSocialistRepublic.htm.

14. South African History Online [Available from: https://www.sahistory.org.za/dated-event/union-south-africa-officially-ceases-exist.
15. Britannica TEoE. Vatican City. *Encyclopedia Britannica* 2021 10 March 2021. https://www.britannica.com/place/Vatican-City.
16. Chiteji FM, Bryceson DF, Ingham K, et al. Tanzania. *Encyclopedia Britannica* 2021 19 March 2021. https://www.britannica.com/place/Tanzania.
17. African National Congress [Available from: https://www.sahistory.org.za/article/african-national-congress-anc.
18. Day J. Southern Rhodesian African Nationalists and the 1961 Constitution. *The Journal of Modern African Studies* 1969;7(2):221-47.
19. Lobban RA, Galli RE, Birmingham D, et al. Guinea-Bissau. *Encyclopedia Britannica* 2022 9 Feb 2022. https://www.britannica.com/place/Guinea-Bissau.
20. Staff R. Taiwan hopes WHO assembly will help boost its profile. *Reuters* 2009 18 May 2009. https://www.reuters.com/article/idUSLI62888 (accessed 4 April 2022).
21. Britannica TEoE. Czechoslovakia. *Encyclopedia Britannica* 2020 12 May 2020. https://www.britannica.com/place/Czechoslovakia.
22. Faris NA, Khalidi RI, Fraser PM, et al. Palestine. *Encyclopedia Britannica* 2021 13 Aug 2021. https://www.britannica.com/place/Palestine.
23. Britannica TEoE. Federation of Rhodesia and Nyasaland. *Encyclopedia Britannica* 2011 6 Jan 2011. https://www.britannica.com/place/Federation-of-Rhodesia-and-Nyasaland.
24. Fürstenau M. East Germany: A failed experiment in dictatorship. *DW* 2019 07 October 2019. https://www.dw.com/en/east-germany-a-failed-experiment-in-dictatorship/a-50717157 (accessed 4 April 2022).
25. Geary PJ, Turner HA, Hamerow TS, et al. Germany. *Encyclopedia Britannica* 2022 30 March 2022. https://www.britannica.com/place/Germany.
26. Miller SG, Laroui A, Swearingen WD, et al. Morocco. *Encyclopedia Britannica* 2022 9 March 2022. https://www.britannica.com/place/Morocco.
27. Oman [Available from: https://www.nationsonline.org/oneworld/oman.htm#:~:text=Oman%20in%20brief&text=The%20oldest%20independent%20state%20in,controlled%20Zanzibar%20and%20other%20territories.

28. Editors Hc. Vietnam War Timeline 2020 [Available from: https://www.history.com/topics/vietnam-war/vietnam-war-timeline.
29. Order of Malta [Available from: https://www.orderofmalta.int/sovereign-order-of-malta/.
30. Britannica TEoE. Pan-Africanist Congress of Azania. *Encyclopedia Britannica* 2017 6 March 2017. https://www.britannica.com/topic/Pan-Africanist-Congress-of-Azania.
31. Patriotic Front (Zimbabwe) [Available from: https://en.wikipedia.org/wiki/Patriotic_Front_(Zimbabwe).
32. Reichard R. Why Isn't Puerto Rico a State? 2021 [updated 4 Oct 2021. Available from: https://www.history.com/news/puerto-rico-statehood.
33. Britannica TEoE. Ruanda-Urundi. *Encyclopedia Britannica* 2008 17 Oct 2008. https://www.britannica.com/place/Ruanda-Urundi.
34. Seychelles Democratic Party [Available from: https://en.wikipedia.org/wiki/Seychelles_Democratic_Party#:~:text=The%20Seychelles%20Democratic%20Party%20is,country%20from%201976%20to%201977.
35. Serbia and Montenegro [Available from: https://www.nationsonline.org/oneworld/serbia_montenegro.htm.
36. Burrowes R, Wenner MW. Yemen. *Encyclopedia Britannica* 2021 10 March 2021. https://www.britannica.com/place/Yemen.
37. Kim H. The American military government in South Korea, 1945-1948: Its formation, policies, and legacies. *Asian Perspective* 1988:51-83.
38. Foster S. Tokelau. *Encyclopedia Britannica* 2018 11 Dec 2018. https://www.britannica.com/place/Tokelau.
39. Klein C. What Countries Were Part of the Soviet Union? 2022 [Available from: https://www.history.com/news/what-countries-were-in-soviet-union2022.
40. Britannica TEoE. United Arab Republic. *Encyclopedia Britannica* 2020 5 March 2020. https://www.britannica.com/place/United-Arab-Republic.
41. Lampe JR, Allcock JB. Yugoslavia. *Encyclopedia Britannica* 2020 5 Nov 2020. https://www.britannica.com/place/Yugoslavia-former-federated-nation-1929-2003.
42. Human Development Reports: Gender Inequality Index (GII): United Nations Development Programme.
